# Genomic evolution and natural history of myeloproliferative neoplasms on therapy

**DOI:** 10.1158/2159-8290.CD-26-0410

**Published:** 2026-09-01

**Authors:** Daniel Leongamornlert, Joe Lee, Aleksandra E. Kamizela, Ken To, Daniel Myers, Nicholas Williams, Kudzai Nyamondo, Xin Wang, Jing Guo, Ruchira K Dissanayake, Jane Price, Amer J Durrani, Jonathan Lambert, Michael Spencer Chapman, John E Pimanda, E Joanna Baxter, Anthony R Green, Anna L Godfrey, Jyoti Nangalia

**Affiliations:** 1 Wellcome Sanger Institute, Wellcome Genome Campus, Hinxton, UK; 2 Lowy Cancer Research Centre, School of Biomedicine, UNSW Sydney, Sydney NSW 2052, Australia; 3 Department of Haematology, University of Cambridge, Cambridge, UK; 4 Cambridge University Hospitals NHS Foundation Trust, Cambridge, UK; 5 University College London Hospital NHS Foundation Trust, London, UK; 6 Queen Mary University of London, Barts Cancer Institute, London, UK; 7 Department of Haematology, Prince of Wales Hospital, Randwick, NSW 2031, Australia; 8 Cambridge Stem Cell Institute, University of Cambridge, Cambridge, UK

## Abstract

Philadelphia-negative myeloproliferative neoplasms are chronic blood neoplasms. Treatments control blood counts but disease can progress to myelofibrosis or acute myeloid leukaemia. We performed longitudinal whole-genome and targeted sequencing in 30 patients, integrating clonal dynamics with 7,986 blood counts and clinical histories. Distinct evolutionary patterns distinguished stable from progressive disease, with leukaemic transformation arising via TP53 loss, stepwise driver mutation acquisition within complex clones, or emergence of independent leukaemic clones. In contrast, stable disease showed long-term clonal equilibrium without new drivers. Phylogenetic analysis using 203 whole-genomes of haematopoietic colonies revealed age-appropriate polyclonal haematopoiesis in triple-negative essential thrombocythaemia and germline predisposition to thrombocytosis, supporting non-neoplastic origins. Therapy-associated mutagenesis was observed, including C>G mutations following azacitidine and characteristic T>A/T>G after hydroxycarbamide exposure in blood cells, although not in skin where UV damage predominated. These findings demonstrate progression is genomically encoded years in advance and support serial monitoring and further study of treatment-related mutagenesis.

## Introduction

Philadelphia-negative myeloproliferative neoplasms (MPN), such as polycythemia vera (PV) and essential thrombocythemia (ET) are chronic haematopoietic neoplasms where both clinical disease and therapy spans decades(1). A major clinical challenge is progression to secondary myelofibrosis (MF) and acute myeloid leukaemia (AML) which occurs in 10% of patients, is often unexpected, and associated with a poor prognosis. While disease transformation is associated with the detection of additional somatic driver mutations, e.g. in *TP53, ASXL1, EZH2* or *IDH1/2*(2), little is understood about the timing of their acquisition prior to overt clinical progression, and whether clinical disease transformation is always genomically encoded. Current clinical management in high risk chronic-phase patients has limited impact on preventing disease progression, instead aiming to limit thrombo-haemorrhagic risk and control blood counts using cytoreduction, most commonly with hydroxycarbamide (HC), as well as non-chemotherapy treatment modalities. However, chronic use of some agents can have long term consequences, such as increased non-melanoma skin cancers (3–6), raising the importance of balancing treatment benefits with risks.

Somatic mutations that drive MPN, such as *JAK2^V617F^*, can occur very early in life(7), followed by lifelong opportunities for genomic evolution, providing a uniquely long window for genomic surveillance and potential intervention during the patient’s journey through MPN. Therefore, a better understanding of the natural history and evolutionary paths to MPN progression, including the consequences of chronic chemotherapy, would enable both earlier identification of high-risk patients and safeguarding the patient population during therapy. Prior longitudinal studies of MPN progression have been restricted to single gene(8–10) or myeloid gene panel sequencing(2,11–13)(bioRxiv 2025.09.23.678057) and have established key routes to MPN progression, including progression mediated by acquisition of additional driver mutations, and the emergence of genetically independent leukaemic clones. However these approaches have limited resolution to define the full spectrum of genomic alterations and clonal relationships underpinning disease evolution. We undertake serial WGS and targeted gene sequencing (TGS) of patients with MPN that either remained clinically stable or developed progressive disease, enabling both temporal and clonal reconstruction of disease trajectories, with the identification of genomic features not captured by targeted genomic approaches. Dynamic genomic architecture was overlaid on serial clinical data to determine how clonal evolution patterns relate to current clinically-defined disease phenotypes. In addition, we applied single-cell derived colony sequencing to reconstruct phylogenetic histories of haematopoiesis to explore the clonal origins of triple-negative ET, and to assess whether chronic chemotherapy leaves detectable mutational footprints in normal and mutant cells. Given the association between long-term cytoreduction and non-melanoma skin malignancies(3–6), we further examined the genomic consequences of MPN therapy on skin epithelium and squamous cell carcinoma in one individual, comparing it to consequences in blood.

## Results

### Patient cohort and longitudinal genomic profiling

**Figure 1:**
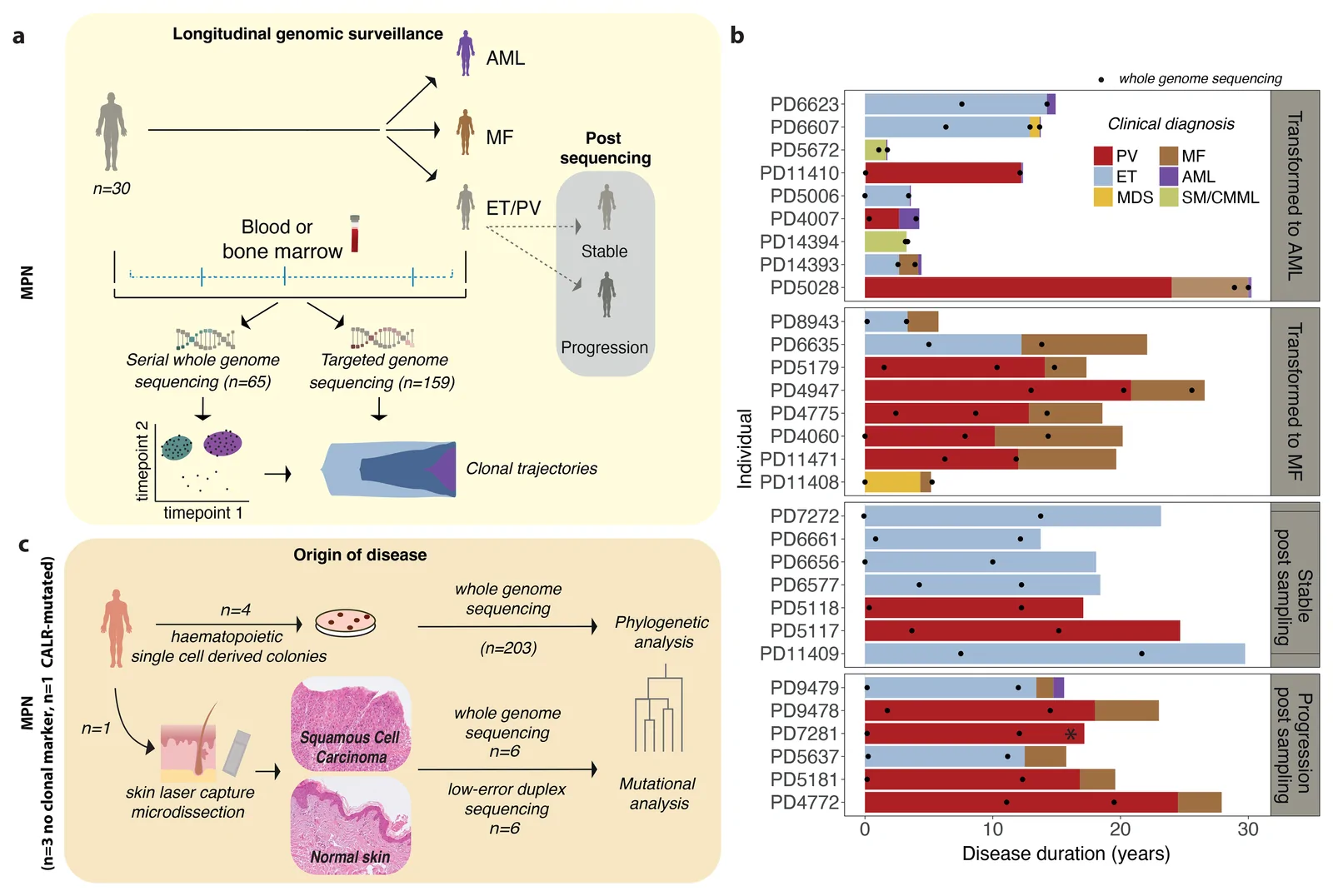
Patient cohort and study design **(a)** Experimental approach. **(b)** Barplot showing the duration of disease from diagnosis for each patient, with colours depicting disease subtype, and points representing samples sent for whole genomes sequencing (WGS). AML, acute myeloid leukaemia; ET, essential thrombocythaemia; PV, polycythaemia vera; MPN, myeloproliferative neoplasm, MDS/MPN, myelodysplastic neoplasm/MPN overlap; SM/CMML, systemic mastocytosis/chronic myelomonocytic leukaemia. **(c)** Experimental approach for haematopoietic coloies and skin tissue.

Serial WGS of blood or bone marrow was undertaken in 30 patients diagnosed at a median age of 58 years (range 33-85 years; Table S1). Patients had chronic phase MPN or MPN/MDS overlap and diverse disease trajectories (Fig.1a,b). Each patient underwent >/=2 WGS timepoints, with a median maximum sampling interval between WGS timepoints of 10.5 years (range 2-174 months). At diagnosis, patients had PV (n=14), ET (n=13), or other chronic myeloid disorders. Sampling spanned chronic and advanced phases, including MF (n=15), AML (n=10), and other myeloid neoplasm states. Clonal architecture was first inferred using longitudinal WGS, integrating both coding and non-coding mutations, and known myeloid drivers. To increase temporal resolution, we performed TGS in 159 additional interval timepoints (median maximum interval 11.4 years, range 13-255 months). Longitudinal clonal dynamics were integrated with clinical diagnosis, treatment history and 7,986 serial blood counts.

Based on clinical course, patients were classified into four groups (Fig.1b): (i) chronic phase MPN progressing to AML (n=9), (ii) chronic phase MPN progressing to MF (n=8), (iii) clinically stable disease throughout sampling and follow-up (n=7); and (iv) stable disease during sampling but progression over subsequent follow-up (MF n=5), AML n=1)). To further interrogate clonal origin and mutation patterns, 203 single-cell derived haematopoietic colonies from four individuals (three ‘triple negative’ ET and one *CALR*-mutated ET) underwent WGS for phylogenetic reconstruction (Fig.1c). In one patient with extensive nonmelanoma skin cancers during long-term MPN therapy, we also performed deep genomic analysis of haematopoietic colonies, healthy skin and squamous cell carcinoma to assess therapy-associated mutagenesis across tissues.

### Accurate somatic mutation identification and deconstruction of clonal composition in MPN

Accurate somatic mutation identification in haematological malignancies is complicated by tumour-in-normal (TiN) contamination, whereby clonal haematopoietic cells are present in matched germline samples. In our cohort, matched germline DNA (purified T cells or buccal swabs) contained variable levels of TiN (0-18%), leading to genuine somatic variants being misclassified as germline and therefore filtered out (Supplementary Fig.1). To address this, we developed TiN-aware somatic variant calling (TiNCan; methods) which estimates the cancer cell fraction in both tumour and matched germline samples and adjusts mutation calling accordingly. Across the cohort, TiNCan rescued the majority of variants that would have been missed by standard callers at 10-20% TiN (Supplementary Fig.2). Using this approach, we identified 56,887 unique somatic mutations (52,889 SNVs and 3,988 indels) across the longitudinal WGS samples, including 394 coding SNVs and 58 coding indels (Table S2). Many myeloid driver mutations were identified, but no recurrent non-coding drivers, including *TERT* promoter variants, were present in this cohort(14–16). The median mutation burden per patient was 1,674 SNVs (range 403–4206) and 129 indels (range 64-339). To reconstruct clonal architecture, we applied Dirichlet-process based clustering(17) to copy number-corrected cancer cell fractions from SNVs (Methods). As each patient had >/= 2 WGS timepoints, mutations assigned to a given clone were required to exhibit coordinated changes in variant allele fractions over time, enabling robust subclonal reconstruction (Supplementary Fig.3) with assignment of coding, non-coding and copy number events to discrete subclones for longitudinal tracking.

### Three routes to AML transformation from MPN

**Figure 2:**
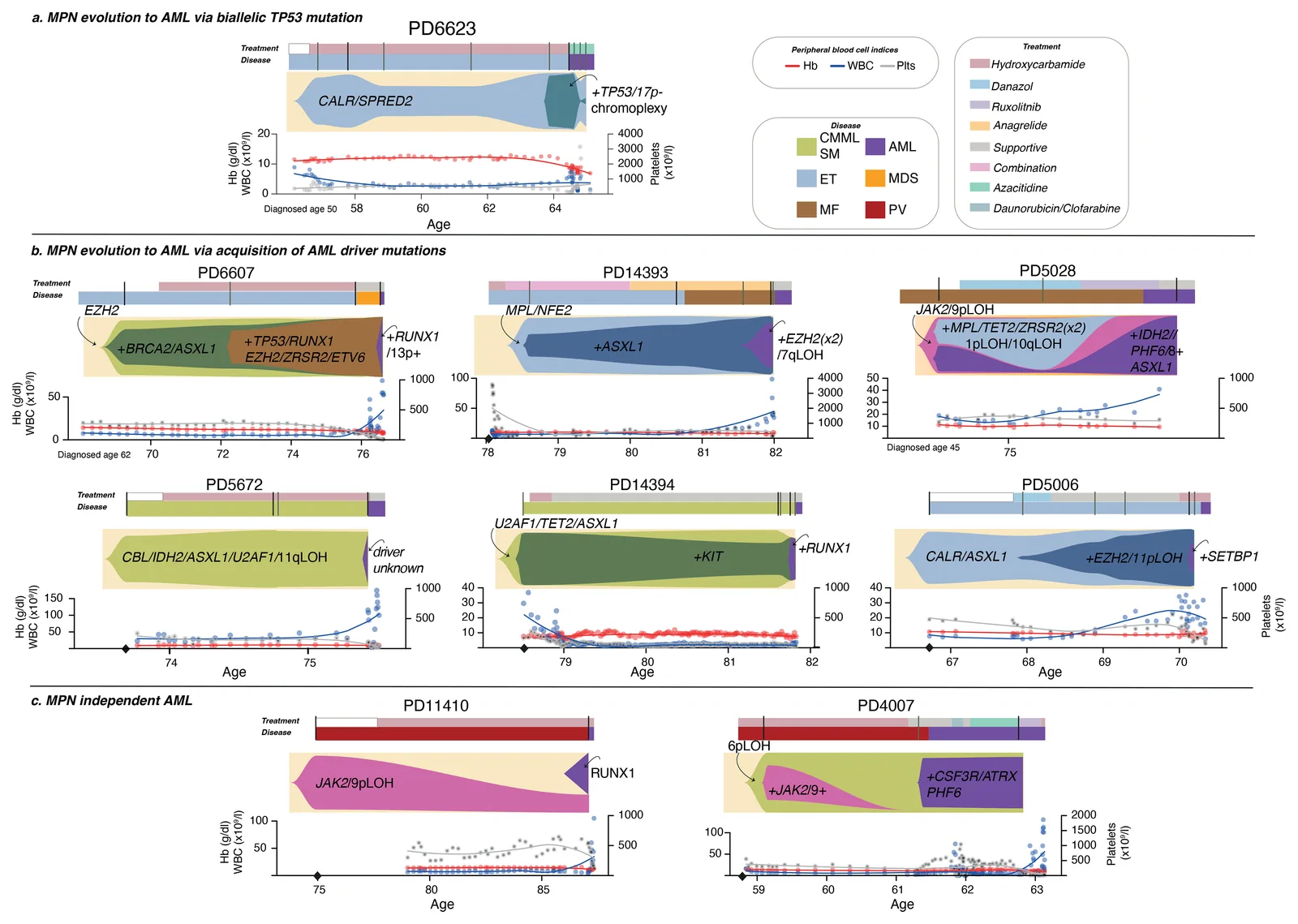
Clinico-genomic integrated vignettes for patients transformed to AML. **(a)** One individual with MPN developing AML through acquisition of *TP53* biallelic loss and subsequent chromosomal copy number changes. **(b)** Six individuals with MPN developing AML via subsequent acquisition of AML driving mutations emerging from advanced MPN clones, often harbouring up to 5 driver mutations. **(c)** Two patients with MPN developing AML that was *JAK2*-negative, showing their independent clonal origin. Disease course is depicted by the top bar coloured by clinical diagnosis. The bar just below shows treatment history coloured by treatment type and indicated as “Unknown” where this information was unavailable. Demarcations between colours demonstrate the time points of initial diagnosis or progression, black lines mark timepoints of samples sequenced with WGS, and grey lines mark timepoints of samples sequenced by TGS. Beneath these are fish-plots showing key clones and subclones with driver events labelled in order of clone appearance. Below this strip, blood counts are shown with the left y-axis for Hb (g/dL) and WBC (x10^9^/L) and the right y-axis representing platelets (x10^9^/L). Trend lines fitted using the geom_smooth(“Loess”) function of ggplot2.

Among nine patients who developed AML, three distinct evolutionary trajectories were observed (Fig.2). **(i)** The first route was through biallelic *TP53* loss (Fig.2a) as previously reported(2,11,13). In one patient (PD6623), AML arose following acquisition of *TP53^R273H^* and subsequent chr17p loss within a *CALR/SPRED2*-mutated clone. The *TP53* mutation was undetectable in four preceding samples (VAF detection limit 0.003-0.059) spanning more than a decade of chronic-phase disease, during which blood counts remained within normal ranges. Within two years of the last *TP53*-negative sample, the *TP53* mutant subclone was fully dominant and accompanied by extensive structural rearrangements shortly before clinical AML diagnosis, consistent with rapid clonal expansion and genomic instability. **(ii)** The most common AML trajectory (6/9 patients) involved sequential acquisition of other AML-associated driver mutations within pre-existing, often heavily mutated, MPN clones (Fig.2b). Antecedent clones harboured mutant-*ASXL1* and accumulated additional alterations including *EZH2, RUNX1, SETBP1, IDH2* and copy number changes. In several cases, low frequency AML-defining mutations were detectable months to years before overt transformation. Dynamic competition between subclones was commonly observed (e.g. PD5028), suggesting differential fitness levels and/or selective pressures during disease evolution. Transformation via this route was typically preceded by antecedent advanced disease (MF or MDS/MF overlap) and characterised by high mutational complexity. **(iii)** Lastly, in two patients with chronic phase PV, AML arose from a genetically distinct clone independent of the *JAK2*-mutated lineage (Fig.2c). In one case (PD11410), AML was associated with emergence of a *RUNX1^Q127X^* clone, and in the other (PD4007), from a clone with *CSF3R, ATRX* and *PHF6* mutations. *JAK2*-negative AML has been previously reported(8–10) but it has been unknown if the MPN and AML shared common ancestry. In PD11410, the AML clone shared only four somatic mutations with the antecedent MPN clone, consistent with a distant common embryonic ancestor, while in PD4007, both *JAK2* and AML clones shared an ancestral 6pLOH clone that arose in utero (~45 mutations of life).

### Clonal dynamics preceding MF transformation

**Figure 3:**
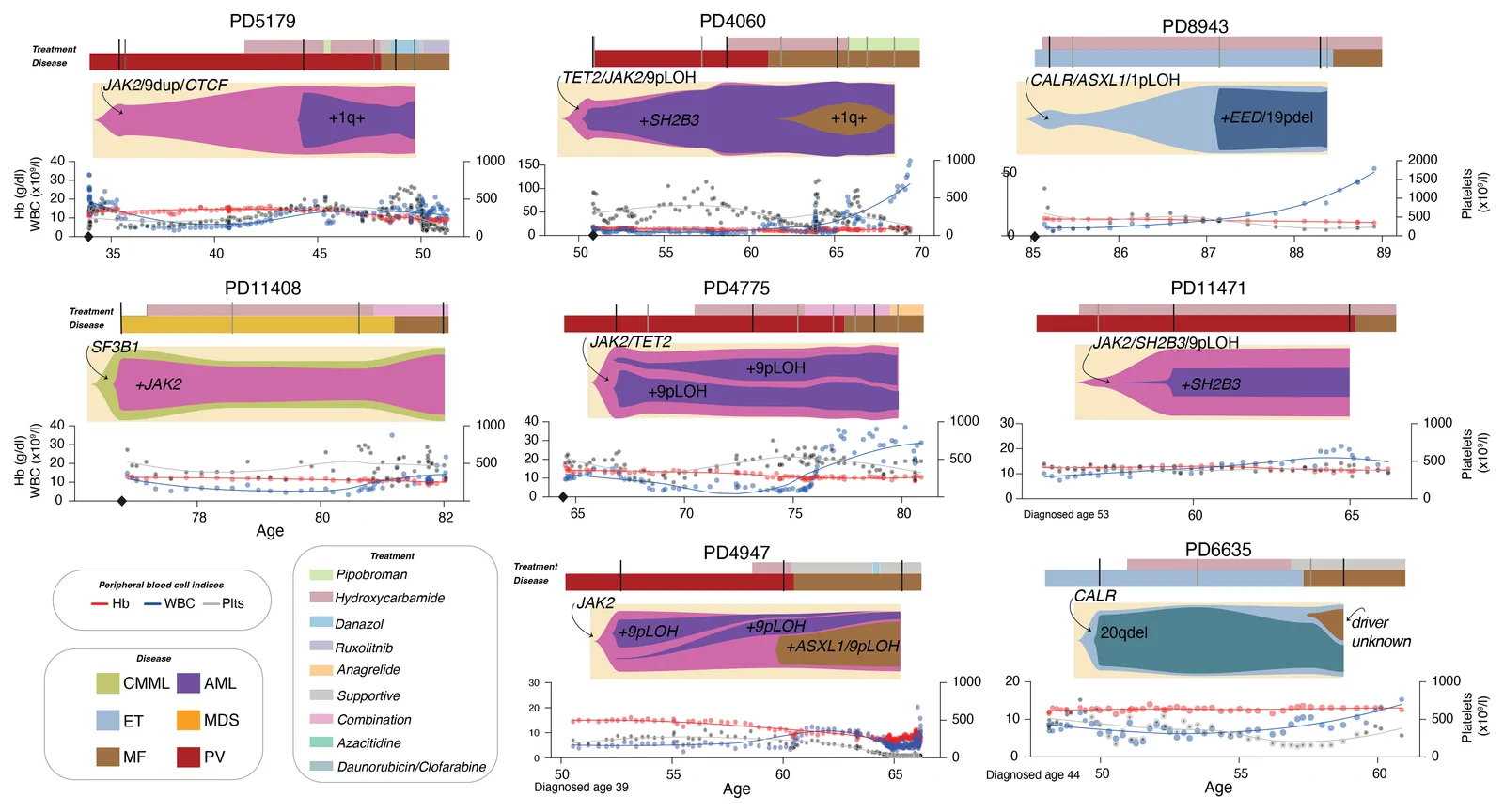
Clinico-genomic vignettes for patients transformed to MF. Eight patients are depicted who have been captured pre- and post MF transformation. Refer to figure 2 for full description of plot features.

Eight patients were sampled longitudinally before and after MF transformation (Fig.3). Across the cohort, all individuals harboured genomically complex disease during chronic-phase MPN, with the dominant clone or subclones already carrying 2 or more additional driver mutations >3-12 years prior to MF diagnosis. In seven patients (Fig.3), MF transformation was preceded by detectable subclonal genomic evolution, characterised by acquisition or expansion of clones with additional drivers. These genomic changes often occurred years before clinical recognition of phenotypic manifestations of disease progression, and similar to those preceding AML transformation (Fig.2), in the context of fully stable blood count parameters. In contrast, in one patient (PD11408, Fig.3), a *JAK2* mutation occurred as a secondary event within an *SF3B1* clone (this individual had refractory anaemia with ringed sideroblasts and thrombocytosis) and this individual did not have any additional post-*JAK2* clonal evolution in the 5 years preceding an MF diagnosis. In some cases, relatively stable, heavily mutated, clones (harbouring up to 4 driver mutations) were observed between chronic phase and MF timepoints over 10 years, without clear emergence of a new dominant driver, suggesting that MF can also develop in the context of gradual phenotypic evolution of existing genomically advanced clones rather than as discrete mutational steps. Changes in blood counts were typically late events prompting repeat marrow evaluation. Our data suggest that MF represents a genomically heterogeneous ‘end-state’ arising through additional subclonal evolution and time-dependent phenotypic progression resulting from existing complex clones.

### Clonal evolution during chronic phase disease

**Figure 4:**
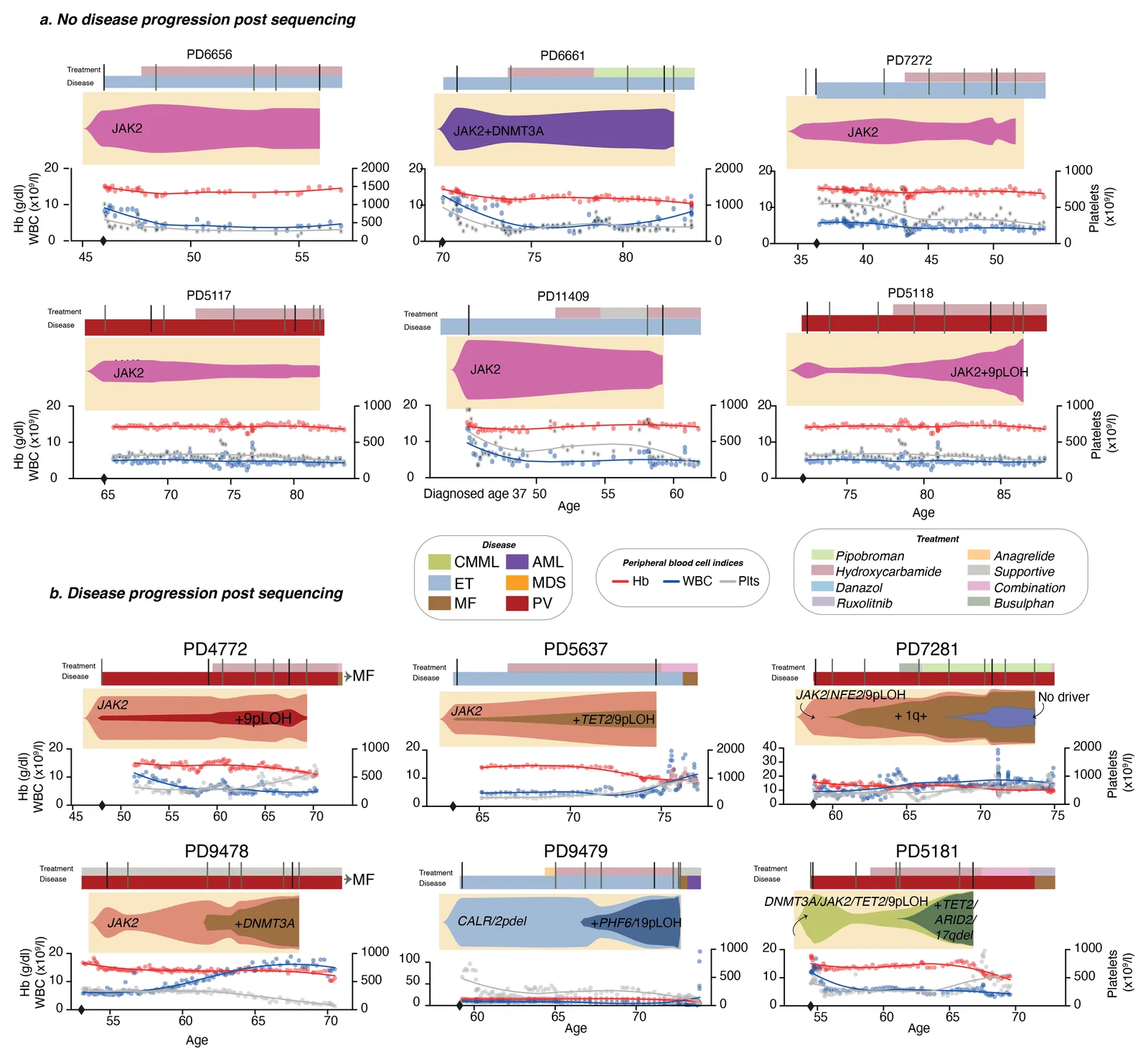
Clinico-genomic vignettes for patients who were clinically stable during samples. Twelve patients sampled during stable disease; six remaining stable **(a)** with subsequent disease progression in six cases **(b)**. PD7281 was suspected to have disease progression with marked leucocytosis (40 × 10^9^/L) and blood count refractoriness to multiple lines of therapy, although progression was not confirmed on bone marrow histology due to patient preference and frailty. Refer to Figure 2 for full description of plot features.

Twelve patients with chronic phase ET or PV with an identified driver underwent longitudinal WGS during clinically stable disease with a median overall follow-up of 18.9 years (range 13.8-29.8) (Fig.4). Six remained clinically stable throughout extended follow-up (Fig.4a), whereas six subsequently developed disease transformation after the final WGS timepoint (Fig.4b). Among patients who remained stable (median follow-up of 8.2 years (range 1.7-9.6) after final WGS sampling), clonal architecture was characterised by long-term equilibrium (Fig.4a). No new drivers or emergent clones were observed during sampling, and clone sizes remained largely unchanged over time. In one case (PD5118), gradual expansion of a *JAK2*-mutated clone occurred despite HC therapy, but without acquisition of additional drivers or phenotypic progression.

In contrast, all six patients who subsequently progressed displayed subclonal evolution with additional driver mutations during their clinically stable phase (median follow-up of 6.3 years (range 2.8-8.5) after final WGS sampling, Fig.4b). In most cases, these subclones demonstrated progressive expansion over time (5-10 years), often acquiring further drivers prior to transformation (PD5181, PD9479). In PD4772, clonal proportions of *JAK2* and 9pLOH were relatively stable over 20 years of sampling during PV. This continued for an additional five years before MF progression during which time we did not have further samples for sequencing so we were unable to assess for subsequent clonal evolution.

Our findings indicate that acquisition of additional driver mutations during chronic-phase disease risks future biological progression, although timelines may still be in the order of many years. Conversely, clonal equilibrium was associated with durable clinical stability.

### Clonal relationships in triple-negative ET

Whether ET that lacks mutations in *JAK2, CALR* and *MPL* (triple-negative ET, TN ET) represents a clonal neoplasm remains uncertain(18–20). Whole exome sequencing and targeted sequencing studies have identified rare germline or somatic *JAK2* and *MPL* mutations in only a minority of cases(2,21). Such patients currently receive a diagnosis of neoplasia despite the absence of a known clonal marker in most cases, and this raises uncertainty over the appropriate use of cytoreduction. To address this, we performed WGS of 181 single-cell derived haematopoietic colonies from 3 patients with WHO defined TN ET (Table S1; mean 13.5x sequencing, range 8-32x, clinical details and histology included in Supplementary Fig.4a,b). Following quality control, 139 (42–49) colonies were included for phylogenetic reconstruction, identifying 101,836 SNVs and 2,798 indels.

**Figure 5:**
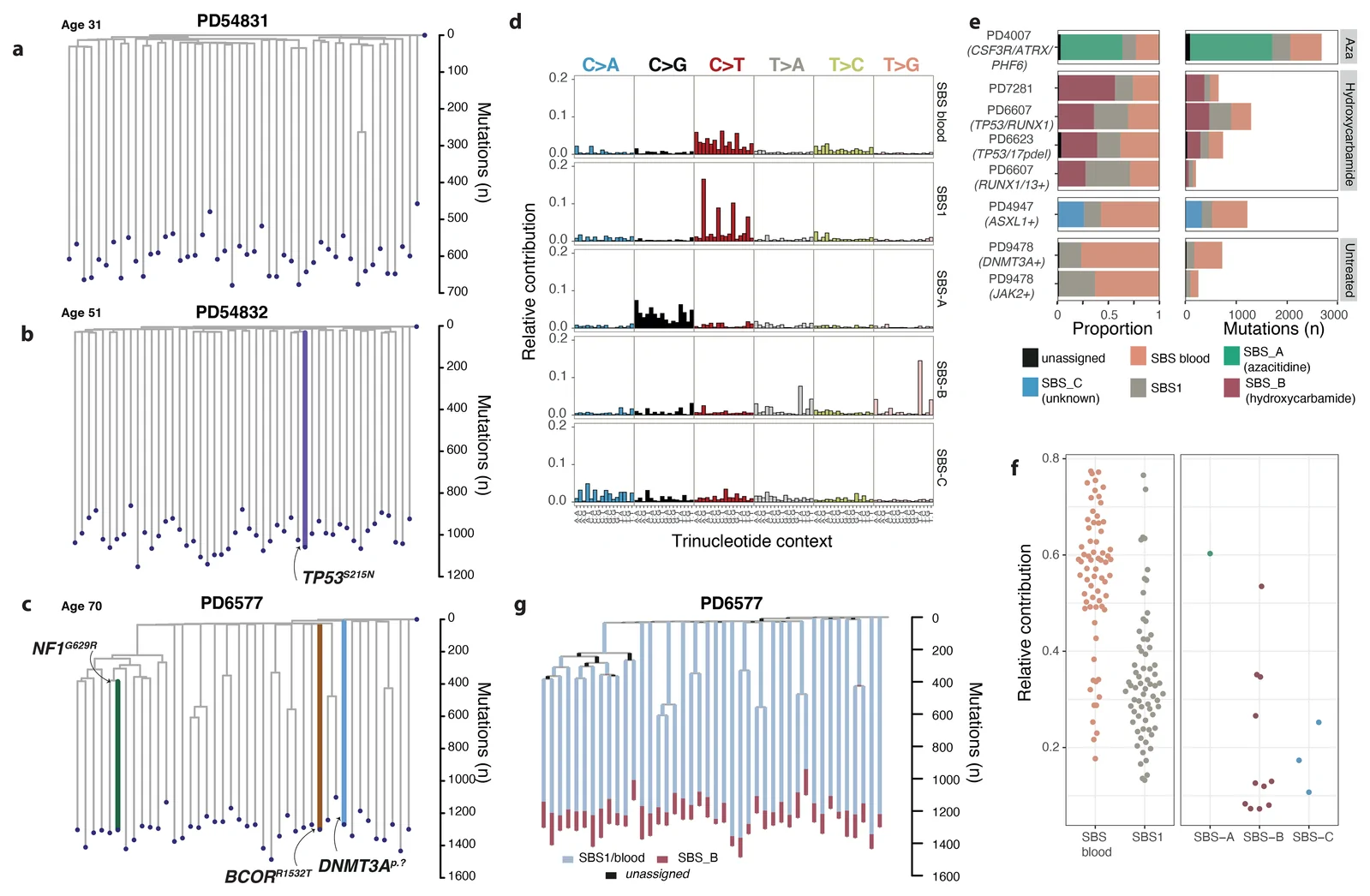
Phylogenetic reconstruction of haematopoiesis and mutational signatures. **(a,b & c)** Single-cell derived haematopoietic colony phylogenetic trees for three individuals with ET lacking mutations in *JAK2, CALR* and *MPL*. Trees describe the pattern of somatic mutation sharing in sampled colonies. The descending vertical axis shows the accumulation of somatic mutation burden from root to tip (top to bottom). The dots at the bottom represent individual sequenced colonies, with their colours corresponding to sampling time points. Highlighted branches are coloured by the driver mutation and copy number change that they carry. Private branches represent those mutations present in only a single colony, and shared branches represent mutations present in the descendant colonies. **(d, e & f)** Shows the results from mutation signature analysis of the WGS MPN cohort using dpclust identified subclones with > 50 SNVs as input. **(d)** 96-trinucleotide context plot of the five signatures identified. **(e)** Stacked barplot showing the relative and absolute contribution of signatures for 6 clones with high non-endogenous signature burden compared to clones from an untreated patient (PD9478). **(f)** Per clone dot plot showing the relative contribution of each signature. Only estimates where the 95% credible interval lower bound was greater than 0 are shown. **(g)** Shows the same phylogenetic tree for PD6577 from **(c)** overlayed with signature attributions per branch.

In two younger patients (PD54831, PD54832), comb-like phylogenies with minimal coalescences were consistent with polyclonal haematopoiesis typical of healthy individuals of similar ages (Fig.5a, b). In one of these patients, a *TP53^S215N^* was detected in a single colony, compatible with small clonal haematopoiesis, either as a result of age or therapy, rather than disease-defining expansion. In the oldest patient (PD6577), several clonal expansions were observed, detectable in single colonies but not in bulk clinical sequencing and therefore, likely at very low VAF. The nature of the driver mutations in this patient (*BCOR^R1532T^, DNMT3A^splice^, NF1^G629R^*) and their occurrence in separate lineages (Fig.5c), combined with the branching architecture of mid-life clonal expansions and normal genome-wide mutation burdens, are typical of phylogenetic findings in this age group that are not associated with thrombocytosis. Overall, all three individuals had phylogenetic trees resembling those expected for their age, in keeping with previously described patterns(22,23). Furthermore, there was no evidence of early-life clonal expansions or sequential clonal expansions characteristic of MPN(7).

Analysis of both somatic and germline whole-genome sequencing data did not identify novel coding mutations of potential pathogenic significance, (Supplementary Table 3), nor mutations associated with inherited thrombocytosis (Supplementary Table 4). We have previously shown that blood cell traits have a strong polygenic germline basis and that TN-ET patients are enriched for those with germline predisposition towards high platelet traits, potentially contributing to their thrombocytosis.(24) Consistent with this, two of the individuals had polygenic germline scores in the top quintile of UK Biobank for platelet counts, with one in the top 5% of scores (Supplementary Fig.5), suggesting that germline-driven platelet production may contribute to thrombocytosis in these cases. Overall, our findings suggest that, in at least a subset of patients diagnosed with TN ET, thrombocytosis may not arise from an underlying neoplastic clone, in keeping with the low risk of disease transformation in such patients. Our data advocate for reconsideration of the diagnostic terminology for such patients in line with our recent UK guidance(25).

### Mutagenicity of hydroxycarbamide and 5-azacitidine in blood cells

Recent genomic studies have demonstrated that several anti-cancer agents - including platinum agents (carboplatin and cisplatin), nitrogen mustard alkylating agents (bendamustine, melphalan, chlorambucil, cyclophosphamide), the triazene procarbazine and antimetabolite 5-fluorouracil - are directly mutagenic to blood cell DNA(26–28). In our cohort, patients were exposed to HC, busulphan, pipobroman, ruxolitinib, anagrelide, danazol and 5-azacitidine (AZA). Although effective for blood count control, the long-term genomic consequences of chronic exposure to these agents is unknown. Both the length of exposure to chemotherapy agents - that can span decades for some agents especially HC - and the heightened risk of secondary skin malignancies (3,6,29), underscores the importance of defining any mutagenicity of current MPN therapies.

Across bulk WGS samples five mutational signatures were identified (Fig.5d). Two corresponded to established endogenous processes in haematopoietic cells: SBS1, characterised by C>T transitions at CpG dinucleotides that accumulate with time and cell division, and SBS Blood, previously described in stem and progenitor cells and of unknown cause(30,31). SBS-A was characterised by C>G transversions across all contexts and was found at high burden in a single individual (Fig.5e & f). This patient (PD4007) was sampled at relapsed AML following recent AZA exposure, where we observed a marked excess (>1000 additional mutations) of SBS-A not present in earlier samples. While AZA associated mutagenesis has not been previously described in humans, similar patterns have been reported in murine models *in vivo* as well as murine and human cells lines, supporting a therapy-associated origin in this sample.(32) Whilst some studies suggest that AZA-induced mutagenesis occurs at CpG dinucleotides in a DNMT1-dependent manner(33), we also observed high mutation burden at non-CpG dinucleotides, consistent with a recent report(32). To validate this AZA-associated mutational signature, we grew haematopoietic colonies in an independent donor with CMML both at diagnosis and following prolonged AZA treatment, confirming the same pattern of C>G transversions in only the colonies following AZA exposure (Supplementary Fig.6).

**Figure 6:**
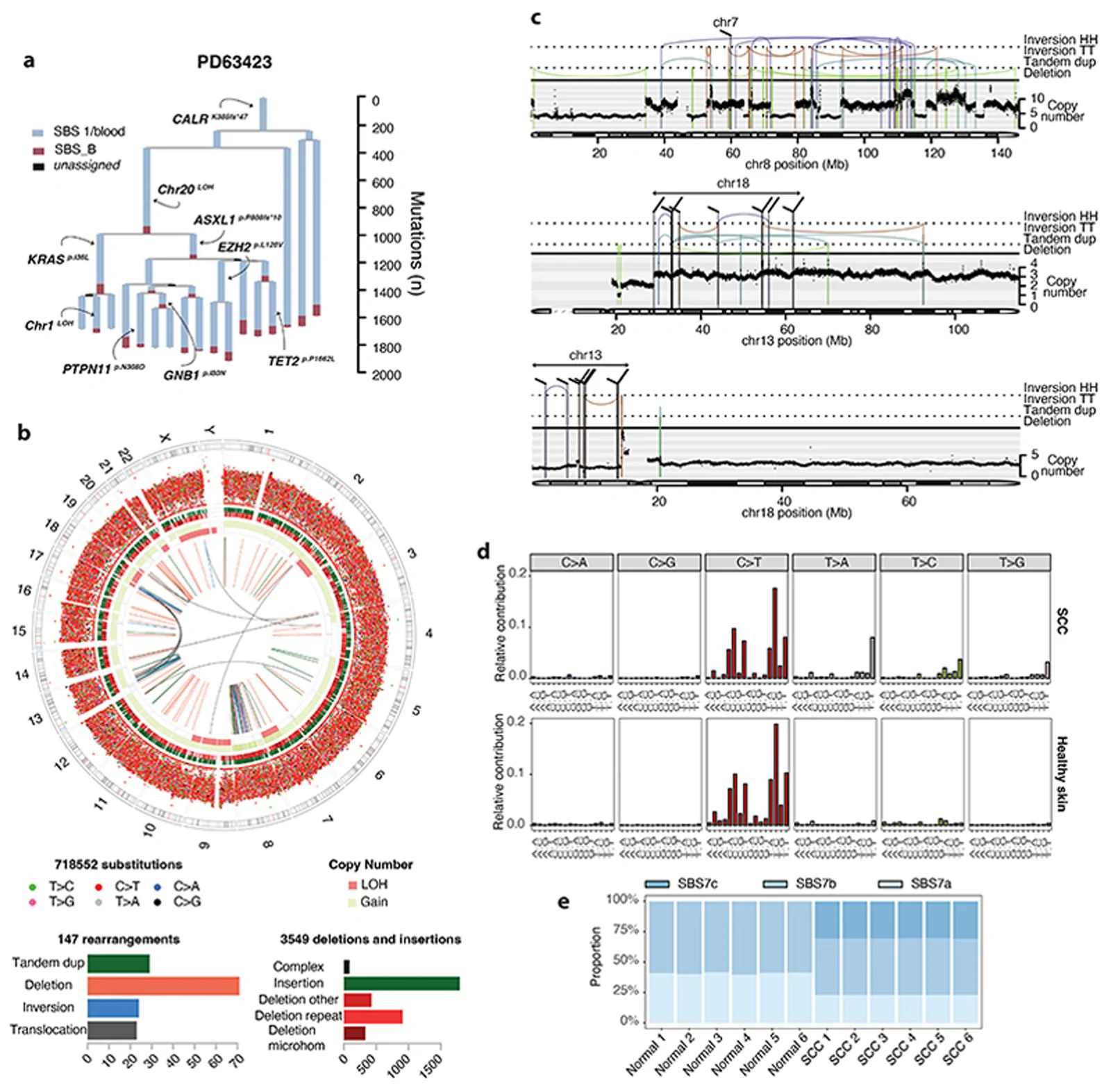
Genomic analysis of blood, healthy skin and squamous cell carcinoma from PD63423. **(a)** Single-cell derived haematopoietic colony phylogenetic tree, overlayed with signature attribution (signature profiles in Extended Figure 5). **(b)** The circos plot shows 4 concentric panels based on data from PD63423c_lo0001; the first shows the chromosome ideogram. The second shows the inter-mutation distance for all SNVs (C>A, blue; C>G, black; C>T, red; T>A, gray; T<C, green; and T>G, pink). The third shows small insertions (dark green) and deletions (red shades) also summarised in a barplot (bottom right). The fourth shows copy number change gains (green) and losses (red). Intra and inter-chromosomal SVs are shown by arcs describing translocations (black), inversions (blue), deletions (red), and duplications (green) and a summary barplot (bottom left). **(c)** Three rearrangement plots from PD63423c_lo0001, the upper showing a highly rearranged chr8 and the lower two multiple translocations between chr13 and chr18. **(d)** A plot showing the 96 context profiles of the cSCC (WGS, n6) and normal (nanoseq, n6) skin epithelium samples. **(e)** Barplot proportions of mutational signatures across healthy and cSCC skin samples.

SBS-B (Fig.5d) was characterised by T>G and T>A substitutions, occurring predominantly at the second thymine of TT dinucleotides. This was reflected in strong peaks at TTT and TTA trinucleotide contexts, indicating a 5’T dependency, with weaker signals at ATA and ATT contexts. In bulk WGS samples, the signature was most prominent in patients who received HC prior to emergence of new clonal expansions, consistent with therapy-induced mutations becoming detectable when carried by expanding clones (Fig.5e). Of such cases, HC-mutations were detected in two AML clones, but the AML drivers themselves did not display the characteristic HC-induced trinucleotide context or mutation. Lower level contributions of SBS-B were observed more broadly across HC-treated individuals (Fig.5f). We confirmed this mutational signature within haematopoietic colonies expanded from single cells in a TN-ET (PD6577, Fig.5g) and *CALR*-mutated (PD63423, Fig.6a, Supplementary Fig.7) patient wherein HC-associated mutations were detectable across every colony (Fig.5g), both mutant and normal haematopoietic lineages, demonstrating that mutagenesis extends beyond tumour clones. Notably, both phylogenies confirmed the absence of this signature in branches predating-HC treatment i.e. not observed on branches <400 mutations of time, (Fig.5g), arguing against selection of pre-existing variants and supporting therapy-associated origin. Lastly, signature analysis also revealed a low level non-specific mutational process (SBS-C) in PD4947 of uncertain cause.

To further validate this HC-associated mutational pattern, we undertook mutational signature analysis of >1000 haematopoietic colonies from our previous study(7). We observed that HC exposure correlated with the presence of HC-induced mutations, cumulative HC dosage, as well as years of exposure (Supplementary Fig.8a-g, HC-mutation number with cumulative HC dose: R^2^ 0.84, p=4.2e-7; HC-mutation number with years of exposure: R^2^ 0.79, p=4.5e-6). Lastly, for one individual (PD5182) in whom colonies were grown both before and after HC exposure, we showed significant increase in HC mutations post-HC exposure (Supplementary Fig.8h-i).

### Analysis of squamous cell carcinoma during MPN therapy

Non-melanocytic skin cancer, such as cutaneous squamous cell carcinoma (cSCC) and basal cell carcinoma (BCC) are an increasingly emergent challenge during MPN therapy, and associated with both HC and ruxolitinib therapy(3,5,6,29). We performed detailed analysis of one MPN patient (PD63423), aged 75, with intractably recurrent BCCs and SCCs. This individual had a 30 year history of *CALR*-mutated ET, 23 years of exposure to HC, several years of treatment with ruxolitinib as well as pegylated interferon, and shorter exposure to anagrelide. In total 9 BCCs and 14 SCCs were resected over 25 years, with the earliest appearing 6 years following commencement of HC. Most, but not all, lesions were on sun exposed skin.

Phylogenetic reconstruction using WGS of haematopoietic colonies (n=22, post-QC n=17) confirmed the expected SBS-B in all blood lineages arising after the 4th decade of life, with no SBS-B in earlier branches, consistent with the individual’s HC treatment history (Fig.6a, Supplementary Fig.7). To determine whether HC similarly contributed to mutagenesis in skin, we performed WGS of a scalp SCC. Given that bulk WGS only captures mutations shared by a larger fraction of cells, thus often representing the mutations in the most recent common ancestor of the clone that may predate HC-exposure, we also undertook ultra-low error duplex sequencing(34) - a technique that allows the detection of mutations within single DNA molecules thus not requiring the presence of a clonal expansion - of normal thigh epithelium to interrogate the mutations present in healthy skin.

All six laser-capture microdissected biopsies of the scalp SCC that underwent WGS were hypermutated, with >700,000 point mutations genome-wide, corresponding to >250 mutations/Mb (Fig.6b). This overall mutation burden is at the upper bound of previously reported cSCC(35) (both sporadic and germline predisposed) and similar to burdens reported in metastatic cSCC(36). We observed complex structural aberrations (147 rearrangements notably affecting chr8, 13, 18, Fig.6c) and mutational signatures associated with UV damage (SBS7a-c, Fig.6d-e). Typical driver mutations associated with SCC were present across all samples and included *NOTCH1, NOTCH2, TP53* and *ARID2*(37–39). No *de novo* signature corresponding to HC was detected in the tumour. To assess whether healthy skin was affected, six laser-capture microdissected biopsies of healthy thigh epithelium from the same individual underwent low-error duplex sequencing. Similarly, no identifiable HC-associated signature was observed in normal skin epithelium (Fig.6e). Given the very high burden of UV-induced damage (tens to hundreds of thousands of mutations per cell), any modest mutational contribution from HC may be masked within the tumour and to a lesser extent the healthy skin samples. We observed that SBS7c contributed a smaller fraction of mutations in the SCC but not the normal skin epithelium (Fig.6e), consistent with prior reports of SBS7c confined to high UV-burden tumours(30,40). These findings suggest that while HC leaves a detectable mutational footprint in haematopoietic cells, its contribution to cutaneous epithelial mutagenesis, if present, is not readily apparent in the context of dominant UV-induced damage, at least in this one individual.

## Discussion

Our findings demonstrate that patterns of genomic evolution in MPN correlate closely with subsequent clinical trajectories. In patients who progressed to MF and AML, genetically defined subclones harbouring additional driver mutations were detectable before changes in blood counts or other clinical features. In contrast, prolonged clonal equilibrium without acquisition of new drivers was strongly associated with clinical stability, even over decades of follow-up.

**Figure 7:**
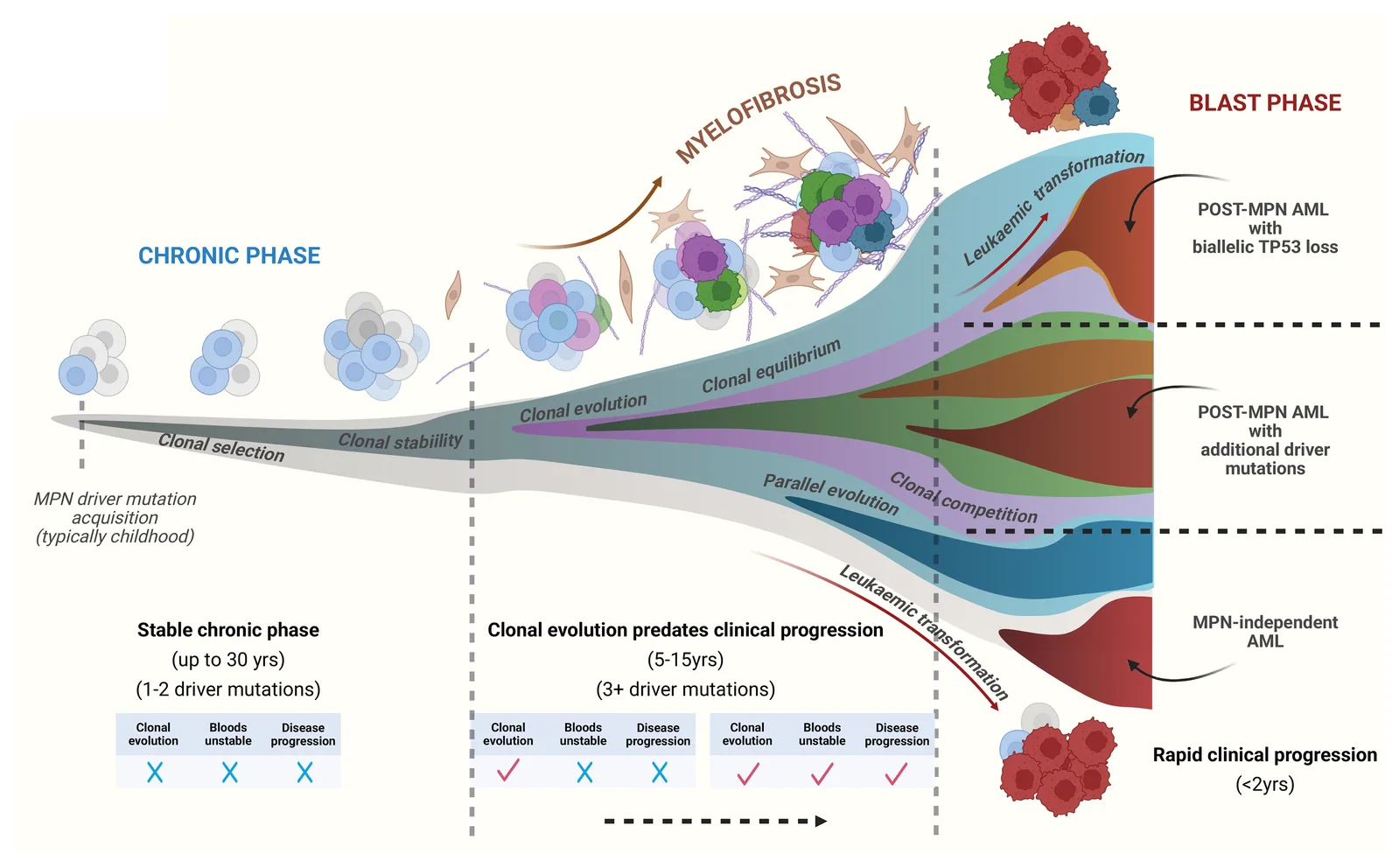
Natural history and evolution of MPN. Illustration of the evolutionary trajectories of MPN from initial driver acquisition.

Taken together with data from prior studies, we can delineate three routes to AML development from MPN: abrupt expansion associated with biallelic TP53 loss; stepwise accumulation of additional driver mutations within already complex MPN clones; and emergence of a genetically independent *de novo* AML clone. The latter observation indicates that AML transformation is not invariably a linear extension of the antecedent MPN clone. Clinical knowledge of the path to AML has implications for both prognosis and therapy, which will require further exploration in larger cohorts. Future clinical studies must establish whether patients with prior MPN but a genetically independent AML are better categorised and managed alongside *de novo* AML, noting that genomic profile is a better predictor of outcome than prior haematological background in other AML subtypes(41). Conversely, AML with biallelic *TP53* inactivation carries a dismal prognosis and patients progressing from MPN should be included in clinical trials of experimental therapies to facilitate allogeneic stem cell transplantation; other patients with older age and/or co-morbidities deserve careful counselling regarding whether to proceed with intensive therapy at all. Patients with low-level and/or monoallelic TP53 inactivation in earlier disease phases would be of particular interest for strategies of enhanced surveillance or novel disease modification. By contrast, progression to MF showed more variable tempo. While all patients harboured genomically complex clones, only a subset demonstrated detectable subclonal expansion in the few years preceding diagnosis, suggesting that MF represents gradual phenotypic evolution of an already advanced clone (Figure 7). Our study is limited by a relatively low number of patients and larger cohorts in the future that reconstruct clonal trajectories from bulk whole-genome sequencing could incorporate these into prognostic modelling and formal risk predictions. Emerging computational approaches that infer clonal histories from bulk sequencing data(42) may enable similar trajectory reconstruction to be applied at greater scale, facilitating integration of evolutionary features into clinical risk prediction models.

Phylogenetic analysis of WHO-defined triple-negative ET challenges the current assumption that all histologically defined ET represents clonal neoplasia. The absence of dominant clonal sweeps or early clonal expansions, and the presence of age-appropriate clonal architectures strongly support the concept that at least a subset of triple-negative ET represents a biologically distinct, non-neoplastic, entity. This has important implications not only for diagnostic labelling of patients, but also therapeutic decision-making, particularly regarding exposure to long-term cytoreduction. Recent guidance, including proposals from the British Society of Haematology (25), has encouraged reconsideration of the triple-negative ET category and adoption of terminology that does not imply an underlying malignancy in the absence of proof of clonal origin.

Antimetabolites such as hydroxycarbamide (HC), and hypomethylating agents, such as AZA, impair DNA replication and epigenetic maintenance. We provide evidence that chronic HC therapy leaves a distinct mutational footprint in haematopoietic cells. HC inhibits ribonucleotide reductase, limiting deoxyribonucleotide availability and inducing replication fork slowing or stalling(43). The dominance of T>G/T>A substitutions at TTT/TTA trinucleotide contexts, suggests preferential mutation of the second T in a TT motif. This pattern could be consistent with sequence-dependent polymerase pausing and misincorporation during replication under dNTP-limited conditions or formation of a sequence-specific DNA adduct. The presence of this signature across all lineages of the phylogenetic tree after HC exposure in an TN ET patient (PD6577), confirms that mutagenesis extends to normal blood cells. This is consistent with our prior observations in children receiving HC for sickle cell disease, although HC-induced mutation burdens in our cohort were higher.(44) We did not detect SBS-B in cSCC or normal skin epithelium from a single individual suggesting that any causal link between HC and secondary malignancies of the skin is not through direct mutagenesis of epithelial cells, and may instead reflect alternative mechanisms such as altered immune surveillance or tumour promotion. However, findings in skin are based on a single individual and therefore require further validation. Furthermore, the absence of functional data limits our ability to address both the underlying mechanism of mutagenesis and potential differences in mutagenicity between cell types.

Unexpectedly, our data revealed substantial C>G mutagenesis in association with AZA exposure in both MPN and CMML patients studied. Observed patterns matched those reported from *in vivo* murine models and both murine and human cell lines(32), but to our knowledge, this has not previously been reported in human samples. Given the widespread use of AZA and decitabine in clinical practice, including in earlier disease states such as cytopenias of uncertain significance, independent validation of this observation is warranted. The clinical significance of the mutations induced by either HC or AZA are currently unknown and characterisation in larger cohorts is warranted with any potential risks balanced against the proven clinical efficacy and safety of these therapeutic agents(45,46). Of note, large population-based and cohort studies consistently show no increased risk of AML or secondary solid organ malignancy with hydroxyurea, and leukemic transformation appears primarily driven by the underlying MPN biology rather than cytoreductive therapy(47,48). Similarly, while preclinical carcinogenicity exists for AZA, evidence for secondary cancers in humans has not been established(49).

Taken together, our data support a shift from current clinical risk stratification frameworks, which in ET and PV remain predominantly focused on thrombosis prevention and in MF centre on survival, towards prognostication that incorporate transformation prediction. Gene panel sequencing is currently not commonly performed in PV or ET in many countries, and is often reserved for transplant planning in MF, typically at a single timepoint. As costs reduce, incorporation of longitudinal targeted sequencing into clinical workflows could refine prognostication, enable earlier identification of high-risk biological evolution, and support more personalised surveillance and intervention strategies. Our study highlights that WGS offers potential advantages over targeted sequencing, providing a comprehensive and unbiased analysis without the constraints of implementing iterative panel designs in a diagnostic laboratory. WGS enables (i) higher-resolution clonal architecture allowing distinction between linear evolution and parallel clonal evolution; (ii) detection and longitudinal tracking of copy-number events currently accessible only through combined myeloid gene panel sequencing with SNP arrays; (iii) identification of therapy-associated mutational signatures; and (iv) interrogation of both germline and non-coding variation in cases lacking canonical driver mutations. These aspects are not accessible through standard targeted sequencing approaches. Overall, the breadth of WGS assessment could in future support confident identification of a group of low-risk patients with MPN and highly stable biology, who could be suitable for long-term low-intensity, remote or “virtual” clinical follow-up. Patients with thrombocytosis lacking any pertinent clonal drivers may be released from long-term specialist follow-up. Delivery of biological risk-adapted therapy remains an aspiration in MPN clinical practice currently but when feasible, the ability to detect and track novel driver mutations could support identification of additional patients whose disease risks an unstable trajectory. Nonetheless, whilst WGS has already been successfully implemented as a routine diagnostic test in specific oncology contexts(50), serial delivery in broad cohorts is currently limited by cost and logistical considerations. In disorders such as MPN, where patients may live for decades through sequential therapeutic exposures, elucidating the mechanisms of secondary malignancies and the cumulative genomic consequences of treatment is essential to ensure long-term blood count control is balanced against late complications.

## Materials and Methods

### Samples

All patients provided written informed consent and the study was undertaken in accordance with the Declaration of Helsinki. The study was approved by the relevant institutional ethics review board. Blood and bone marrow samples were obtained from patients recruited from Addenbrooke’s NHS Foundation Trust under the Causes of clonal blood cell disorders study (07/MRE05/44). Triple negative ET samples and SCC tissue samples were obtained under the Cambridge Blood and Stem Cell Biobank ethics protocol (REC ID 18/EE/0199). Clinical data regarding the patients including blood counts and treatment history were collected using the electronic health record (EHR) systems. Whole blood and skin biopsies were obtained during routine clinical visits with buccal swab or T-cell samples (following *in vitro* culture to reduce myeloid cell contamination) from some patients for matched ‘normal’ material.

### Isolation of mononuclear cells, granulocytes and CD34-positive cells from fresh blood samples

Mononuclear cells (MNCs) were isolated from whole blood by first diluting 1:1 with PBS and undergoing density gradient separation using LymphoprepTM (STEMCELL technologies) gradient separation technique. The MNC fraction was then collected and washed with PBS followed by red cell lysis by incubating in RBC lysis buffer (Biolegend). Following MNC fraction collection, the remaining plasma and Lymphoprep were removed leaving the granulocyte sediment which was resuspended in PBS and subsequent red cell lysis. CD34+ cells were selected from MNCs using the EasySep human whole blood CD34 positive selective kit (STEMCELL Technologies) as per the manufacturer’s instructions.

### DNA preparation and sequencing of ‘bulk MPN’ samples

DNA extraction using the QIAmp DNA extraction kit (Qiagen) was carried out on MNCs, granulocytes, T-cells, cultured T cells and buccal epithelial cells as per the manufacturer’s instructions. DNA samples were submitted for whole-genome sequencing library preparation on Illumina HiSeq 2500 platform with 100bp paired-end reads.

### Somatic mutation calling in bulk whole-genome sequencing (WGS)

Somatic variant calling requires both a tumour sample and a “normal” sample, in haematological malignancies this is complicated by tumour contamination in matched normal samples which will be referred to as ‘Tumour in Normal’ (TiN) from here on. Initial variant calling was performed using the in-house pipelines developed as part of the cancer genome project (CGP) by the CGP IT team in the Cancer Ageing and Somatic Mutations (CASM) department at the Sanger Institute. Samples were aligned to the human reference genome (NCBI build37) using BWA-MEM (https://github.com/lh3/BWA). Matched tumour/normal variant calling was performed to identify Single nucleotide variants (SNVs), using CaVEMAN (https://github.com/cancerit/cgpCaVEManWrapper), Short Insertions and deletions (Indels) using cgpPindel (https://github.com/cancerit/cgpPindel), copy-number aberrations (CNA) using ASCAT (https://github.com/cancerit/ascatNgs) and Battenberg (https://github.com/cancerit/cgpBattenberg) and structural variants (SVs) using BRASS (https://github.com/cancerit/BRASS). Additionally HATCHet (https://github.com/raphael-group/hatchet) was used where multiple subclonal copy number events were suspected and *TERT* promoter mutations were screened using bcftools mpileup (https://github.com/samtools/bcftools).

### Single nucleotide variant calling in the context of TiN contamination (CaVETiN)

A modification to the CaVEMAN algorithm, termed CaVETiN, which is able to account for the degree of TiN was developed. The TiN value can be explicitly specified in the CaVETiN algorithm. For each single base genomic locus, the CaVEMAN algorithm seeks to identify single base substitutions by exhaustively calculating the probability of each possible germline and tumour genotype that is consistent with a separately specified locus-specific chromosomal copy number for germline and tumour cells. Typically for humans, autosomal loci will have 2 chromosomal copies in a normal sample and perhaps a variable copy number for a tumour sample. Standard CaVEMAN has a user-provided parameter, called “contamination”, that represents the estimated proportion of normal cells in the tumour sample. An alternative parameterisation would be to specify the aberrant cell fraction, ^*α_t_*^, instead (contamination=1 − ^*α_t_*^).

In standard CaVEMAN the normal sample is assumed to have an aberrant cell fraction of zero, whereas in our TiN aware approach the normal sample is treated in the same way as the tumour sample with its own estimated aberrant cell fraction, ^*α_n_*^. For a given sample, tumour or normal, the likelihood of the sequence data at a site can be expressed as the product of the probability of the observed called base at each of the sample reads that overlap the site given the germline and tumour genotypes and the aberrant cell fraction. Then for each of these reads the probability of the observed base call is calculated whilst accounting for sequencing error and the probability that the read arose from a tumour or germline cell (reflecting the aberrant cell fraction) and then, given the hypothesised genotype of cell, incorporates the probability that the read is from a mutant or reference chromosome (reflecting the relative number of mutant alleles in the genotype). The details of the above calculation for the tumour sample and the standard CaVEMAN algorithm are detailed in Supplementary note 1. Benchmarking performed at various TiN levels confirmed that in most samples a TiN value of 0.1 or 10% tumour in the matched normal sample was sufficient to allow recovery of genuine variants in our cohort. For the final SNV call set we used a fixed 10% contamination in CaVETiN.

### Insertion/deletion calling in the context of TiN contamination

Indels were called by our standard in-house indel caller (Pindel). To recover variants confounded by TiN contamination, variants initially removed due to presence in the matched normal (F015 flag) were recovered and then refined by performing Germline log odds (GLOD) filtering as described previously in Williams et al. 2022(7).

### Driver identification

Coding drivers were identified by using previously identified myeloid malignancies and clonal hematopoiesis drivers (Grinfeld 2018, Papaemmanuil 2013, Tazi 2022, Pich 2022, Bernstein 2024, Bick 2020).

### Subclonal reconstruction of the tumour compartment

Reconstruction of the subclonal compartment was achieved by using the dpclust algorithm (https://github.com/Wedge-lab/dpclust), a Bayesian clustering method using a Drichlet process. This method utilises the copy number corrected cancer cell fraction (CCF) for variants. Per patient all WGS samples were processed using dpclust3p (https://github.com/Wedge-lab/dpclust3p), using SNVs from CaVETiN and copynumber from Battenberg.

### Bait set design for targeted sequencing

A custom bait set was designed using the Agilent Sureselect software including all coding SNVs and indels from all samples and 10% of non-coding mutations in each identified subclone. Samples underwent hybridization- based pull-down and standard library preparation for Illumina sequencing on the Novaseq platform to a mean depth of 1350x (128-2056x). Two samples with <500x depth were removed from further analysis.

### Clinico-genomic integrated vignettes

The disease course is depicted by the top bar coloured by clinical disease diagnosis alongside therapy received for MPN. Demarcations between colours demonstrate the time points of initial diagnosis or progression, black lines mark timepoints of samples sequenced with WGS, and grey lines mark timepoints of samples sequenced by TGS. Beneath these clinical phenotype/sample time-point bars, fish-plots showing key clones and subclones with driver events labelled in order of clone appearance, (see detailed description below). Below this strip, blood counts are shown with the left y-axis for Hb (g/dL) and WBC (x10^9^/L) and the right y-axis representing platelets (x10^9^/L). Trend lines fitted using the geom_smooth(“Loess”) function of ggplot2. The bottom bar-plot shows treatment history coloured by treatment type and indicated as “Unknown” where this information was unavailable.

### Fishplot construction

To be able integrate both WGS and TGS samples onto a single fishplot (https://github.com/chrisamiller/fishplot), we calculated CCF as double the median VAF of diploid autosomal SNVs assigned to a clone. TGS samples were dropped if duplicates of another included timepoint. Where the TGS callable variants were insufficient to track a clone of interest we reverted to plotting just the WGS samples. Coding drivers and copy number events were checked and allocated to clones manually. In one patient (PD4007) the root cluster containing 6pLOH was recovered using copy number data from WGS samples and therefore the CCF for this clone was inferred for the interstitial TGS sample. As our subclonal reconstruction is limited by sample purity and sequencing depth, we have endeavoured to maximise the information content of these plots by assigning any unallocated drivers (from clones below our sensitivity) to the most likely parental clone.

### Haematopoietic colonies

Four patients underwent single-cell expansion of collected MNCs in Methocult for subsequent WGS. Freshly prepared or thawed isolated MNCs were diluted in PBS to a concentration of 6-7 x106 cells/ml. 200μl of this cell suspension was added to 5.5mL aliquots of MethoCult H4034 Optimum (STEMCELL Technologies) which had been prepared at room temperature. Following vortex mixing, each aliquot was divided into approximately 1.5mls per well using a syringe and an 18-gauge needle. Plates were incubated at 37°C with 5% CO2 for approximately 14 days at which point they were visualised under a microscope. Individual colonies were identified based on morphology and erythroid (BFU-E) colonies were manually picked into 50μl of RLT lysis buffer (Qiagen) and stored at -80°C.

### DNA preparation and sequencing

10-20μl of lysed colony suspensions (20-45% of each colony) were submitted for library preparation via the ‘laser capture microdissected biopsy’ (LCMB) whole genome sequencing (WGS) pipeline [24] at the Wellcome Sanger Institute (WSI) core facilities with 8 cycles PCR. This pipeline allows the generation of high complexity WGS libraries from an input of 150-200 cells, such as those present in a single colonic crypt or endometrial gland. The normal LCMB pipeline (for solid tissue biopsy samples) usually utilises 12 cycles of PCR, however, for my colonies, I only required 8 cycles of PCR due to the higher quantities of DNA present compared to LCMB. Only samples with over 2ng/μl of generated library DNA were used for WGS (150bp reads, paired-end) on NovaSeq machines, as samples at <2ng/μl would generally suffer from higher duplicate rates. Reads were mapped to the human reference genome (built GRCh37, NCBI) using the BWA-MEM (Burrows-Wheeler Aligner) algorithm.

### Mutation calling in haematopoietic colonies

Single nucleotide variants (SNVs) were identified using the CaVEMan algorithm using an in silico normal sample (PDv37is). CaVEMan was run on the following parameters: mutant copy number: 5, wild type copy number: 2, normal contamination: 0. Small insertions and deletions (indels) were called using cgp-Pindel algorithm [26]. Structural variants were called using BRASS pipeline [27]. Both cgpPindel and BRASS used PDv37is as an unmatched normal sample.

### Phylogenetic reconstruction

Construction of phylogenetic trees used methods previously described in Williams et al. 2022(7) and are briefly described below. Following detection of somatic mutations, mutations shared between all colonies from a single patient are treated as germline variants and excluded. Subsequently, using VAFcorrect a data matrix of the number of reads supporting every mutation, depth of the sequencing of that site, and the variant allele fraction, across every colony for a patient was constructed. A data matrix is then used to generate a matrix of genotypes where each mutation is assigned a status: ‘1’, if the mutation is present, ‘0’, if absent, or NA (unknown) based on the VAF of the mutations and the depth of sequencing at that site in every colony. The structure of phylogenetic trees is then inferred using MPBoot which utilizes a maximum parsimony approach. This method minimizes the number of changes required to reach the set of mutations assigned to each sample. This algorithm only uses the genotypes of SNVs to derive the topology and branch lengths of the phylogenetic tree, however both SNVs and indels are then placed onto tree branches. Both private and shared branch lengths are then adjusted for the varying depths of sequencing across colonies.

### Tissue methods

Standard LCM was used to sample both normal and SCC skin tissue. The standard LCMB pipeline was undertaken to isolate from LCM as described in Ellis at al. 2021. The normal skin samples were then sent to the WGS Nanorate sequencing pipeline while the SCC tissue was sent for Standard LCMB-WGS pipeline.

### Signature analysis

SNV signature analysis was performed using HDP (https://github.com/nicolaroberts/hdp) to extract de novo mutational signatures (with the following parameters burnin=10000, spacing=500, n_per_chain=250), with the input matrix formatted with 96 rows describing the SNV contexts and each column the cluster or branch-level count. The extracted signatures were assessed for similarity to known mutational signatures in the COSMIC database v3.2 and SBSblood, a signature attributed to the endogenous mutational processes in HSPCs with a cosine similarity matrix using the MutationalPatterns (https://github.com/UMCUGenetics/MutationalPatterns) package which was also used to plot the mutational signatures. Signature analysis was performed separately for MPN bulk cohort and colony data sets.

### Skin samples

Skin biopsies were placed into 70% ethanol or PAXgene FIX, following which they were put into standard histology cassettes before being paraffin embedded and trimmed and cut to the desired thickness. For staining, paraffin was removed using xylene, 100% ethanol, 70% ethanol and deionised water washes, followed by Haematoxylin and eosin staining. Whole-slide imaging was performed before and after laser-capture microdissection cutting using the NanoZoomer S60 digital slide scanner. Regions of interest were outlined and annotated on scanned images of the permanently covered Superfrost specimen slides and the temporary cover-slipped PEN slides using NDP View 2 software. Following laser calibration, areas of cSCC and healthy epithelium were cut into a 96-well plate, followed by protein digestion using the Arcturus PicoPure DNA extraction kit prior to library preparation for either whole genome sequencing or whole-genome nanoseq.

### Circos plot

The circos plot was made with RCircos (https://github.com/hzhanghenry/RCircos), using SNV (Caveman), indel (pindel), CNV (ASCAT) and SV (BRASS) data. The plot shows 4 concentric panels; the first shows the chromosome ideogram. The second shows the intermutation distance for all SNVs (C>A, blue; C>G, black; C>T, red; T>A, gray; T<C, green; and T>G, pink). The third shows small insertions (dark green) and deletions (dark red). The fourth shows copy number change gains (green) and losses (red). Intra- and interchromosomal SVs are shown by arcs describing translocations (black), inversions (blue), deletions (red), and duplications (green).

### Rearrangement plots

Rearrangement plots use the outputs of BRASS to show both copy number and intra and inter chromosomal SV events. The bottom part of the plot shows the absolute copy number from the brass output *.ngscn.abs_cn.bg, the vertical lines describe different SV types; deletion (D; bright green), tandem duplication (TD; dark green), head to head inversion (HH; purple) and tail to tail inversion (TT; dark orange) from the brass output *.brass.annot.bedpe. Intra-chromosomal events are joined by arcs at the higher (HH & TT) or lower (TD & D) dotted lines for their respective events, inter-chromosomal events are annotated with the partner chromosome.

## Supplementary Material

Supplementary Figure S1

Supplementary Figure S2

Supplementary Figure S3

Supplementary Figure S4

Supplementary Figure S5

Supplementary Figure S6

Supplementary Figure S7

Supplementary Figure S8

Supplementary Note 1

Supplementary Tables S1-S4

## Data Availability

Code for analyses will be made available and deposited at https://github.com/nangalialab/MPNevolution. Sequencing files have been deposited in the European Genome–Phenome Archive (https://www.ebi.ac.uk/ega/home) with accession codes EGAD00001016067 (MPN bulk WGS), EGAD00001016066 (TN-ET colonies) and EGAD00001016068 (PD63423 colonies, LCMB-WGS and nanoseq) in line with the Wellcome Sanger Institute data sharing policy, and all somatic mutation.vcf files will be uploaded to Mendeley (DOI: 10.17632/hsz7nkwxgy.1).

## Statement Of Significance

Longitudinal whole-genome sequencing shows myeloproliferative neoplasm progression is genomically encoded years before clinical transformation, with distinct evolutionary routes to leukaemia and myelofibrosis. It identifies DNA mutagenesis associated with hydroxycarbamide and 5-azacitidine, suggests some triple-negative cases are non-clonal, and supports serial clinical genomic monitoring for improved risk stratification and long-term management.
