# Supplementary material for: Genomic evolution and natural history of myeloproliferative neoplasms on therapy
Source: Cancer Discov. Author manuscript; Available in PMC 2026 May 15. (PMC7619087; doi:10.1158/2159-8290.CD-26-0410)
Supplement: Supplementary Figure S1 [file EMS213397-supplement-Supplementary_Figure_S1.pdf]

Supplementary Figure 1. The presence of 'tumour-in-normal' contamination

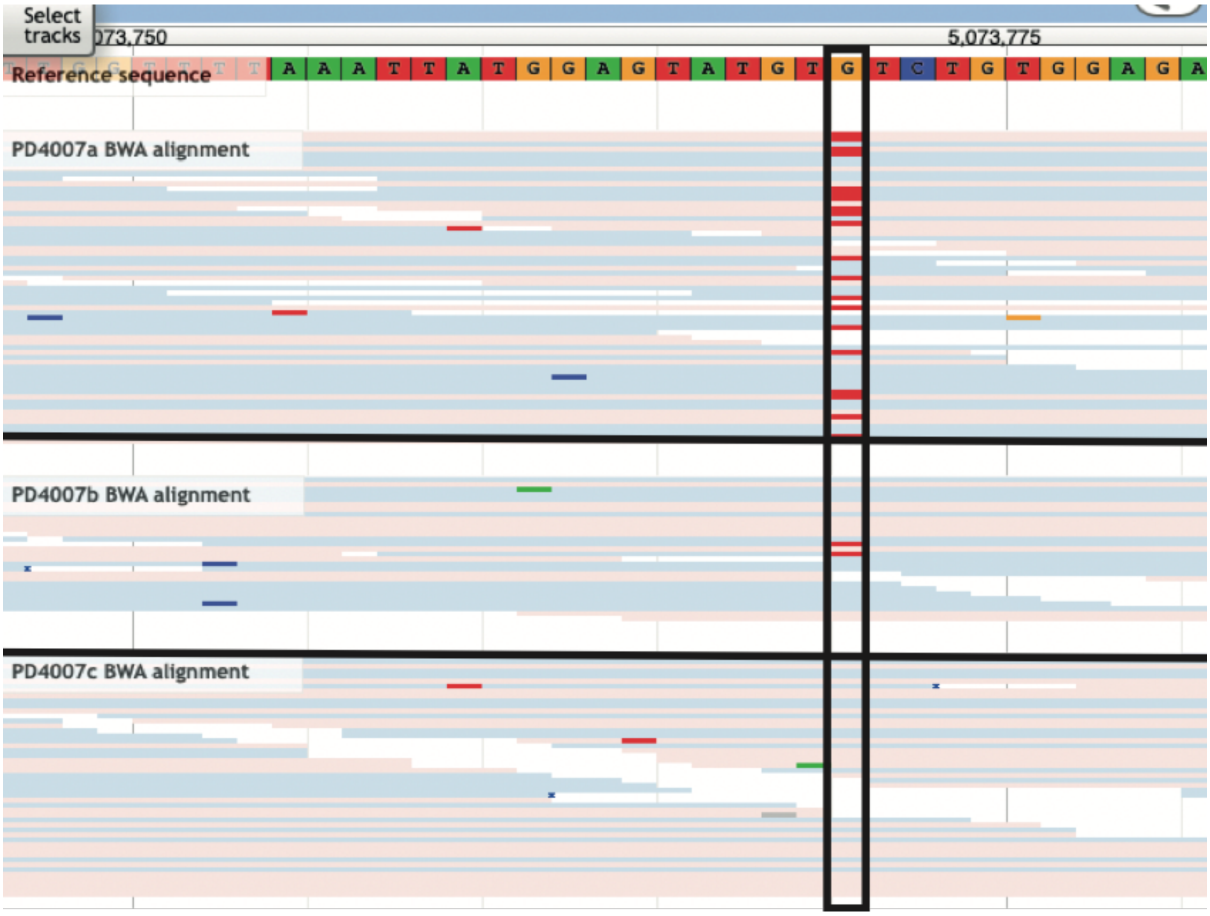

Supplementary Figure 1. Jbrowse plot of PD4007a (tumour first time point), PD4007b (germline, buccal epithelium DNA) and PD4007c (tumour second time point) samples showing the G>T transversion at *JAK2* (V617F) (shown by the red bars in the highlighted black box). Two red reads are seen in the germline sample which results in the mutation in the tumour being assigned as a germline mutation using standard variant callers. Note the absence of red reads in the second time point tumour sample, confirming the loss of *JAK2*^{V617F} clone. Reads coloured by strand, blue = top strand, red reads = bottom strand.
