# Supplementary material for: Genomic evolution and natural history of myeloproliferative neoplasms on therapy
Source: Cancer Discov. Author manuscript; Available in PMC 2026 May 15. (PMC7619087; doi:10.1158/2159-8290.CD-26-0410)
Supplement: Supplementary Figure S2 [file EMS213397-supplement-Supplementary_Figure_S2.pdf]

Supplementary Figure 2. TiNCan somatic mutation caller

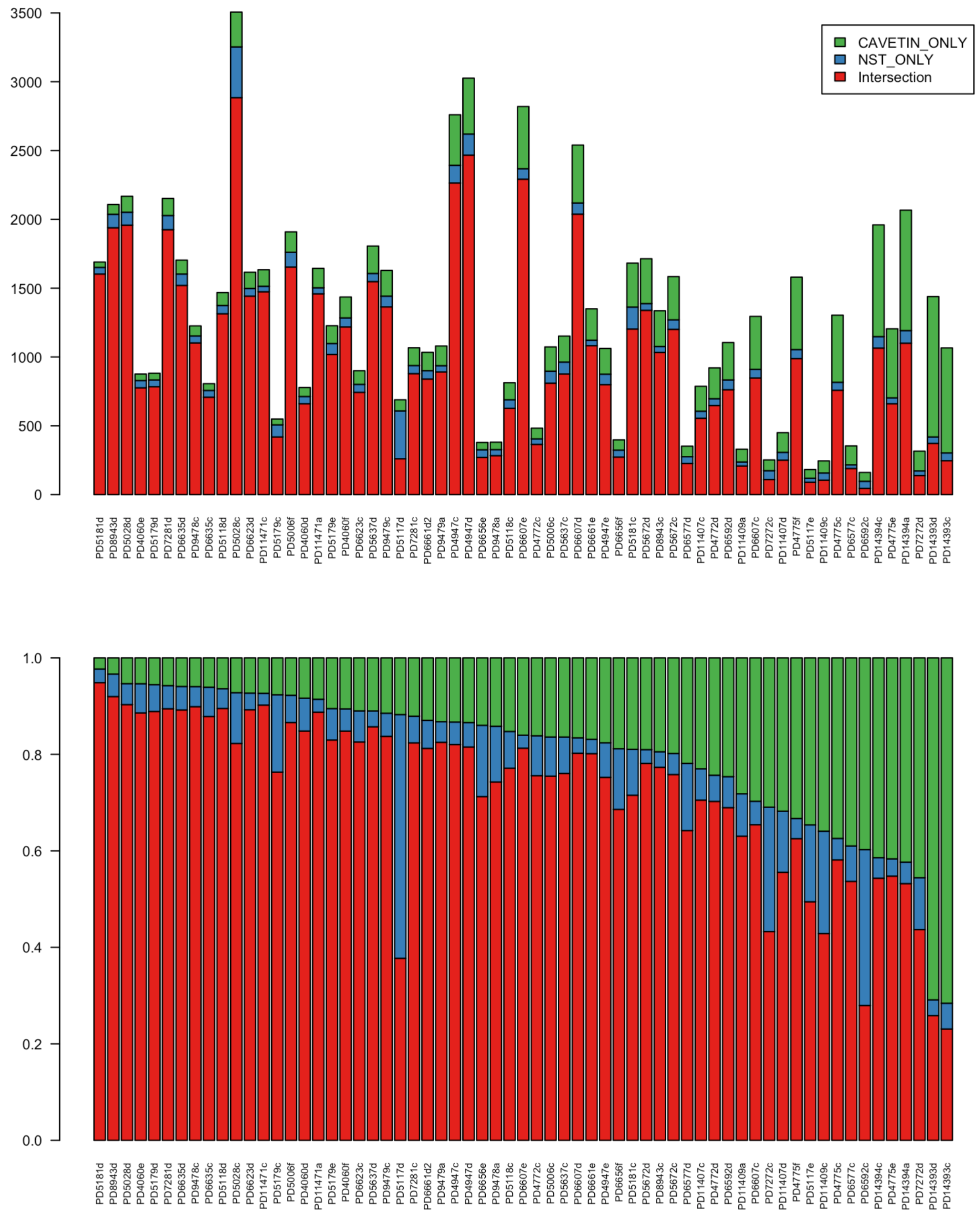

Supplementary Figure 2. Barplots showing variant recovery by TiNCan compared to standard caller. Top stacked barplot describes absolute counts of SNVs categorised by caller overlap (intersection, TiNCan-only or NST(standard CaVEMaN-only). Bottom stacked barplot shows SNVs categorised normalised by total count.
