# Supplementary material for: Genomic evolution and natural history of myeloproliferative neoplasms on therapy
Source: Cancer Discov. Author manuscript; Available in PMC 2026 May 15. (PMC7619087; doi:10.1158/2159-8290.CD-26-0410)
Supplement: Supplementary Figure S3 [file EMS213397-supplement-Supplementary_Figure_S3.pdf]

Supplementary Figure 3. Assigning somatic mutations to tumour clones and subclones

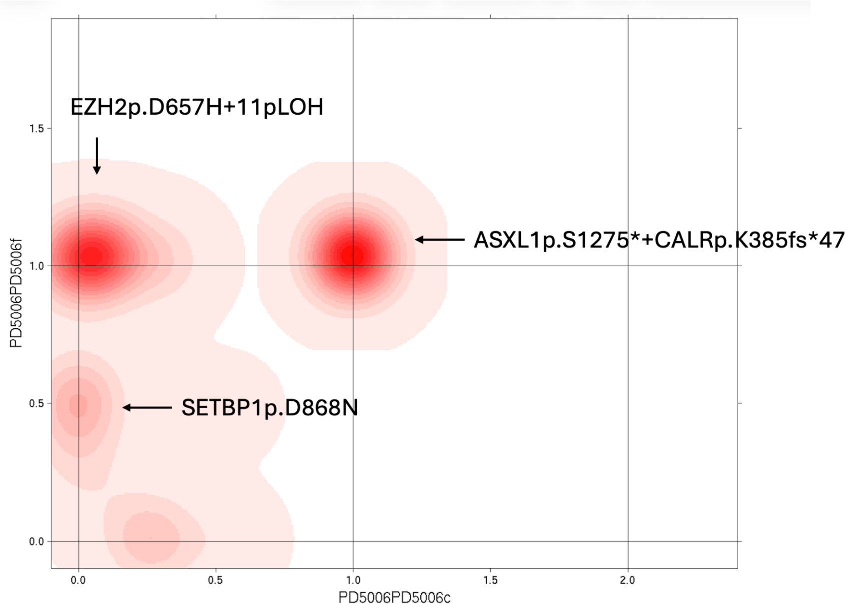

Supplementary Figure 3. An example of clustering of mutations to individual clones and subclones by dpclust for PD5006. The algorithm uses samples from multiple time-points to allow for accurate assignment of mutations to cancer clones based on the trajectory of the cancer cell fraction (CCF) of each variant. CNVs and indels are then manually annotated to the clones based on the clone that has a CCF that is closest to the CCF of the CNV and indels.
