# Supplementary material for: Genomic evolution and natural history of myeloproliferative neoplasms on therapy
Source: Cancer Discov. Author manuscript; Available in PMC 2026 May 15. (PMC7619087; doi:10.1158/2159-8290.CD-26-0410)
Supplement: Supplementary Figure S4 [file EMS213397-supplement-Supplementary_Figure_S4.pdf]

Supplementary Figure 4. Histology and clinical details of Triple-negative ET patients

a.

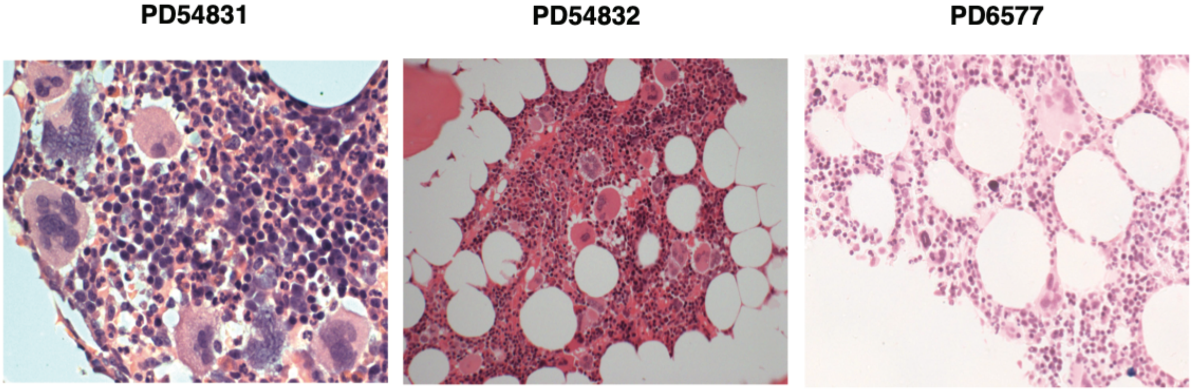

b.

Clinical information

Patient	Gender	Age at diagnosis	Age at sample	Plts at sample	Treatment
PD54831	F	30	31	1383	Pegylated interferon
PD54832	F	51	51	1038	Aspirin
PD6577	F	58	71	229	Anagrelide (5 years), Hydroxyurea (12 years), Aspirin

Supplementary Figure 4. (a) Bone marrow biopsy pictures from PD54831, PD54832 and PD6577. (b) Summary of clinical history from PD54831, PD54832 and PD6577 including age at diagnosis, age at sample, platelet count (plts) at sample and therapy received.
