# Supplementary material for: Genomic evolution and natural history of myeloproliferative neoplasms on therapy
Source: Cancer Discov. Author manuscript; Available in PMC 2026 May 15. (PMC7619087; doi:10.1158/2159-8290.CD-26-0410)
Supplement: Supplementary Figure S5 [file EMS213397-supplement-Supplementary_Figure_S5.pdf]

Supplementary Figure 5. Germline polygenic scores (PGS) for four platelet traits in the three TN-ET patients.

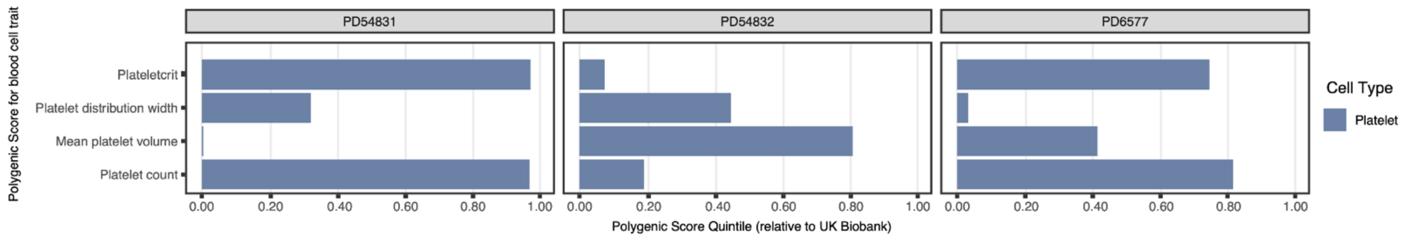

Supplementary Figure 5. The x-axis shows the PGS quintile (based on the distribution of scores in UK Biobank). PD54831 has a very high PGS for Platelet count and Plateletcrit (top 5% of UK Biobank) and similarly, PD6577 is in the top 20% of UK Biobank for germline determined high platelet count. This is not the case for PD54832, but this individual has germline-determined high mean platelet volume, the relevance of which is unknown.
