# Supplementary material for: Genomic evolution and natural history of myeloproliferative neoplasms on therapy
Source: Cancer Discov. Author manuscript; Available in PMC 2026 May 15. (PMC7619087; doi:10.1158/2159-8290.CD-26-0410)
Supplement: Supplementary Figure S6 [file EMS213397-supplement-Supplementary_Figure_S6.pdf]

**Supplementary Figure 6. Mutation signatures in haematopoietic colonies from one CMML individual (PD7151) pre and post AZA exposure**

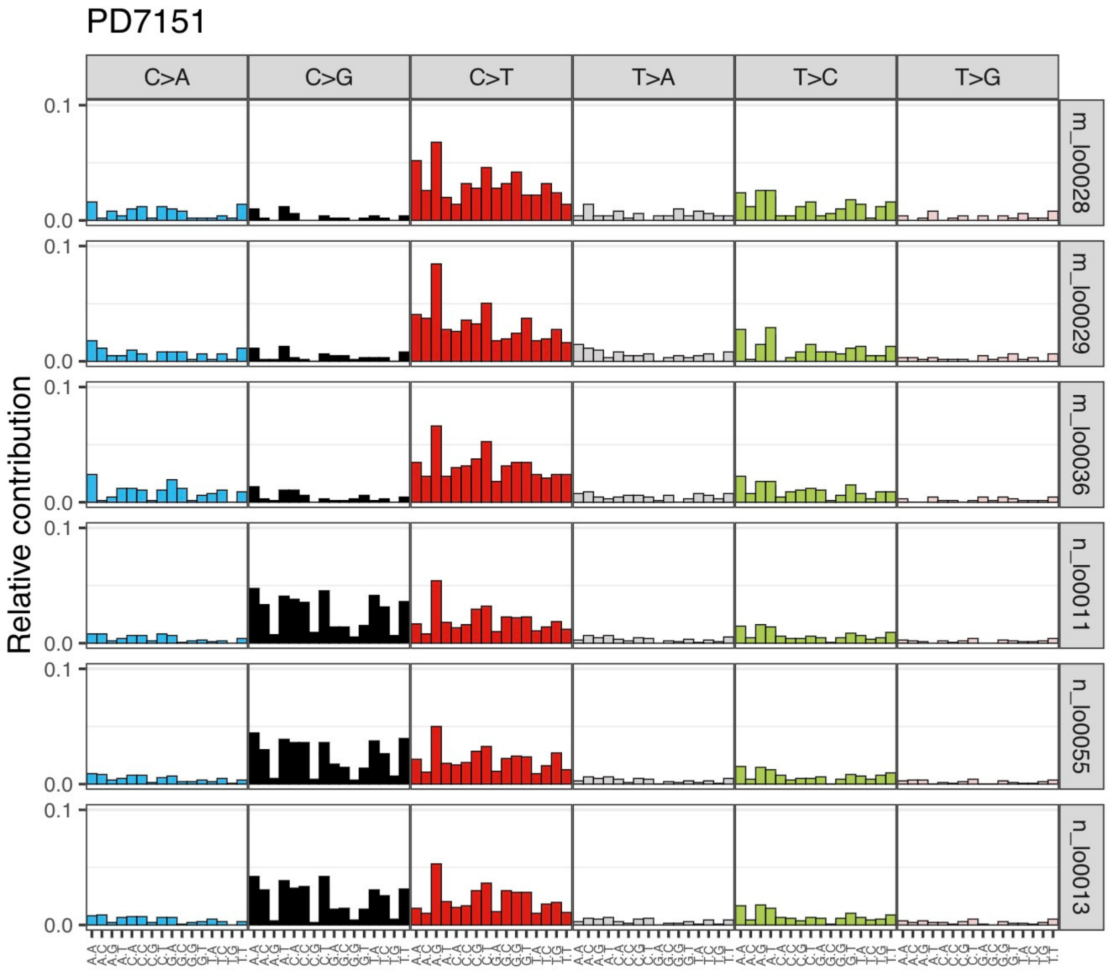


**Supplementary Figure 6.** Trinucleotide context of mutations present in six haematopoietic colonies from PD7151 with CMML. Timepoint 1 (top 3 rows, M_lo028, M_lo029 and M_lo036 are from pre-AZA). Timepoint 2 (bottom 3 rows, N_lo011, N_lo055 and N_lo013) are from after 145 cycles of AZA given over a 12 year period since the first timepoint.
