# Supplementary material for: Genomic evolution and natural history of myeloproliferative neoplasms on therapy
Source: Cancer Discov. Author manuscript; Available in PMC 2026 May 15. (PMC7619087; doi:10.1158/2159-8290.CD-26-0410)
Supplement: Supplementary Figure S7 [file EMS213397-supplement-Supplementary_Figure_S7.pdf]

Supplementary Figure 7. 96 context mutational signature profile from PD63423 haematopoietic colonies

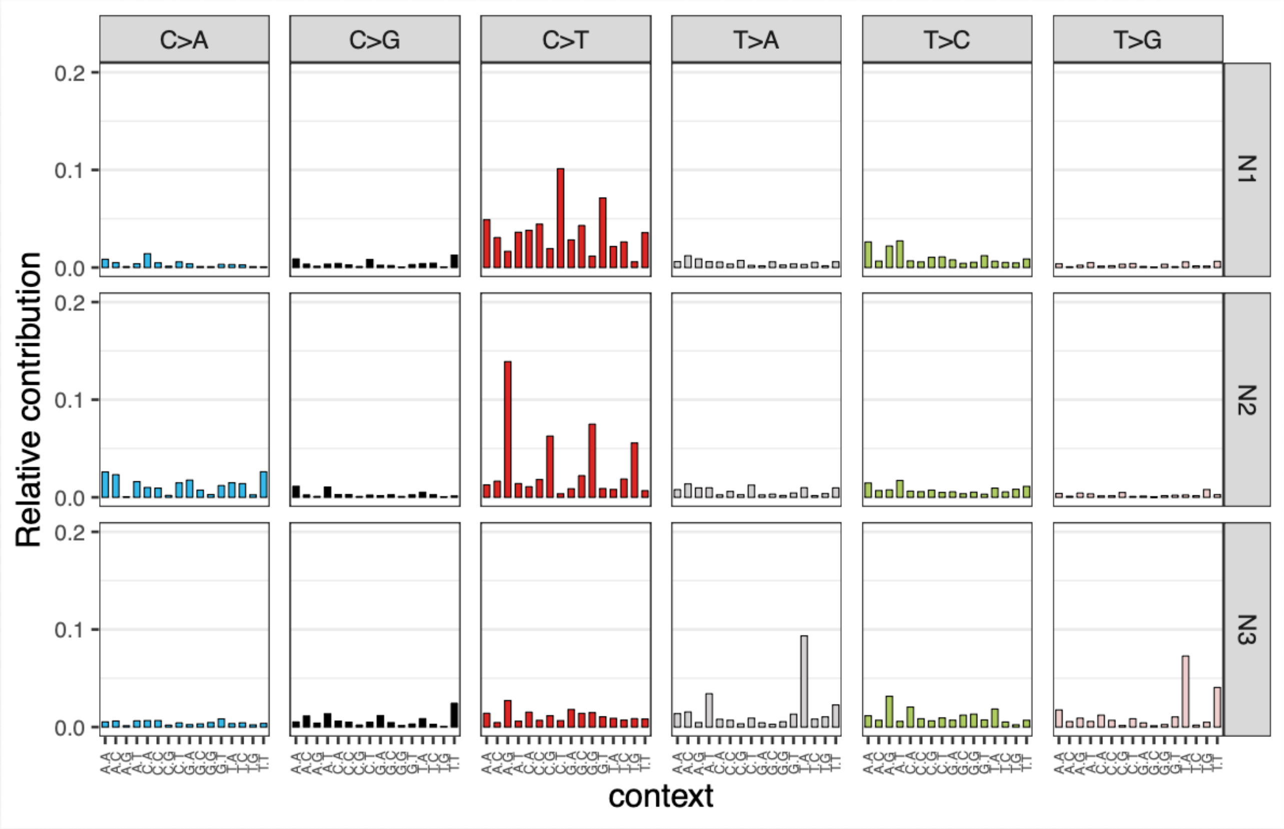

Supplementary Figure 7. A plot showing the 96 context profiles of haematopoietic colonies from PD63423 showing 3 signatures. N1 (SBS Blood), N2 (SBS1) and N3 (Hydroxycarbamide signature, referred to as SBS-B in Figure 5d-f).
