# Supplementary material for: Genomic evolution and natural history of myeloproliferative neoplasms on therapy
Source: Cancer Discov. Author manuscript; Available in PMC 2026 May 15. (PMC7619087; doi:10.1158/2159-8290.CD-26-0410)
Supplement: Supplementary Figure S8 [file EMS213397-supplement-Supplementary_Figure_S8.pdf]

Supplementary Figure 8. HC-associated mutations in Williams *et al* Nature 2022.

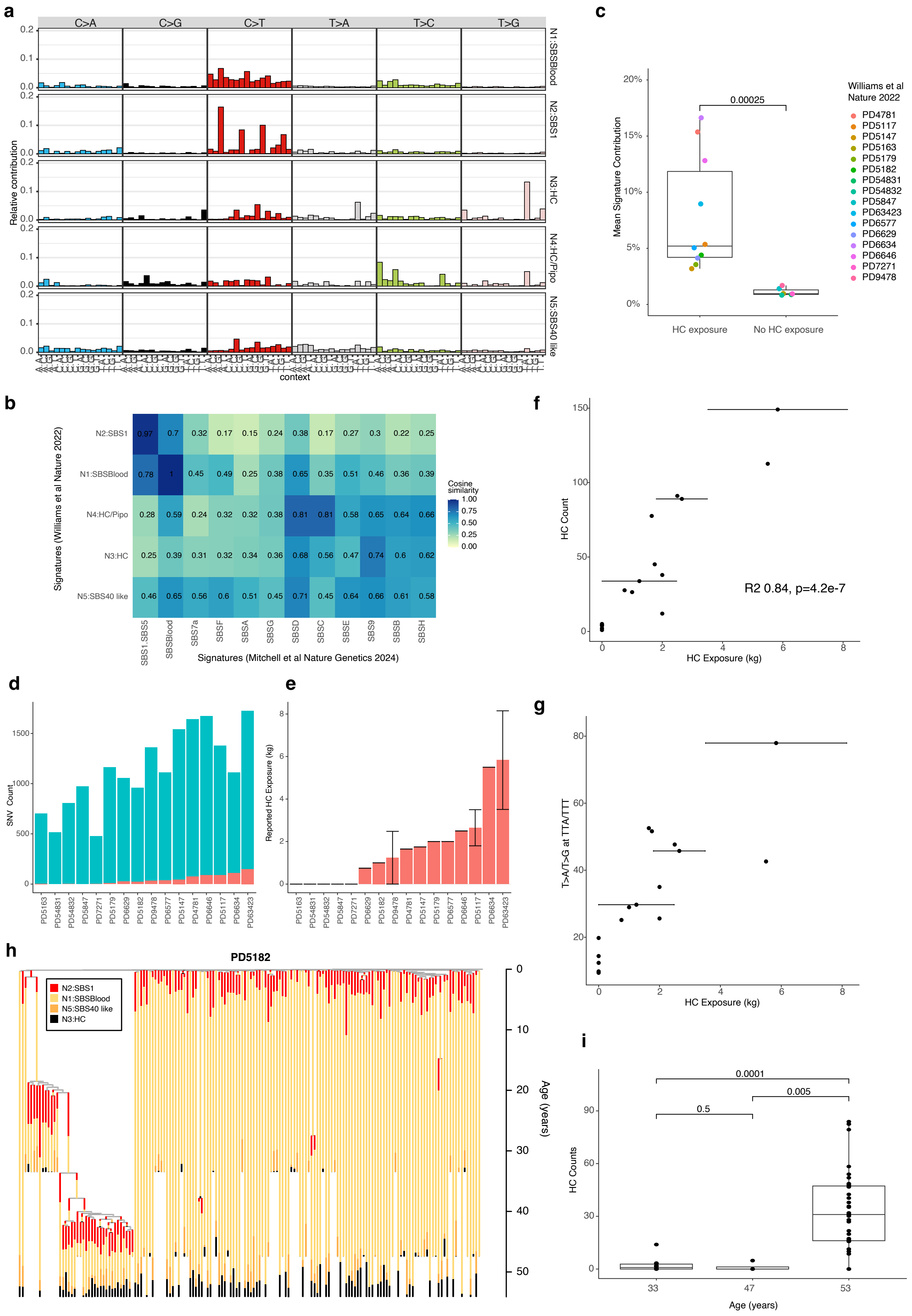

Supplementary Figure 8. De novo signature analysis of >1000 colonies from 12 donors (Williams et al, Nature 2022) extracted 5 signatures (**a**) – N1, SBS Blood; N2, SBS1; N3 showing the characteristic pattern of HC as observed in SBS-B in our current study; N4 which appears to be a mixture of the HC signature and alkylator-associated mutagenesis in an individual receiving both HC and pipobroman, and N5 which was SBS40 like. **b.** Cosine similarities of the 5 signatures extracted to those reported in Mitchell et al Nature Genetics 2024. Note the similarity between N4 and the SBSD and SBSC (0.81 cosine similarity) signatures reported in association with alkylators in that study. **c.** The mean signature contribution of N3 across all branches of the phylogenetic trees of the donors is shown by HC exposure. P value, Wilcoxon Rank Sum. **d.** Number of HC associated mutations (red) compared to total SNVs (blue) averaged across all colonies per donor. **e.** Reported HC exposure in total kg per donor. Error bars reflect uncertainty over information on dosage missing for some periods. **f/g.** Number of HC associated mutations (**f)** or specific T>A/T>G at TTA/TTT mutations (**g**) by HC-exposure (kg). **h.** Phylogenetic tree of PD5182 who was sampled at two timepoints prior to HC exposure and one timepoint after HC exposure showing HC associated mutations in black. **i.** Comparison of number of HC associated mutations by different timepoints. Age 33 and 47 timepoints are pre-HC exposure. Age 53 is after HC. P value, Wilcoxon signed-rank
