# Supplementary material for: Genomic evolution and natural history of myeloproliferative neoplasms on therapy
Source: Cancer Discov. Author manuscript; Available in PMC 2026 May 15. (PMC7619087; doi:10.1158/2159-8290.CD-26-0410)
Supplement: Supplementary Note 1 [file EMS213397-supplement-Supplementary_Note_1.docx]

**Supplementary note 1. CaveTiN Formulation**

In this short note we introduce a generalization of the CaVEMan algorithm to accommodate Tumour-In-Normal contamination. At each site of the genome CaVEMan calculates the probability of there being a somatic mutation given a pre-specified aberrant cell fraction
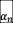
 and
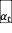
 of the normal and tumour sample respectively, a site specific copy number for both germline,
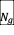
, and tumour,
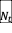
, and also prior probabilities that the site has a somatic variant,
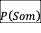
, or germline variant,
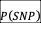
.

At each of these sites the model considers the set of possible germline,
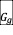
, and tumour genotypes,
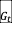
, constructed from the reference allele
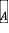
 and a single variant allele
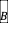
. We have the following disjoint sets of allowed joint genotypes:


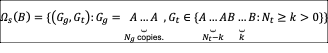


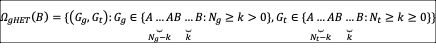


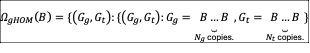


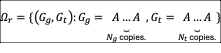


Of course, the variants allele
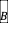
 can be any of the 3 non-reference alleles. Thus, the joint genotypes consistent with somatic and germline variation with a single variant allele are :


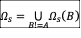


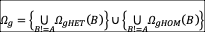


The union of the 3 sets
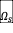
,
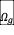
 and
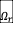
 give the set of possible genotypes,
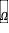
 considered by the model. We can then express prior probability of set membership probabilities in terms of the caveman priors
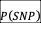
 and
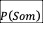
:


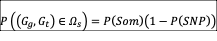


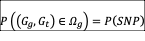


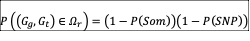


Note the above assumes that somatic and germline variants are mutually exclusive.

We assume a flat prior within each of the above sets - let
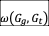
 represent which of the above subsets of
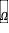
 that
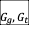
 belong to, then:


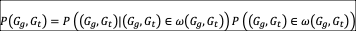


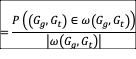


Where we’ve already calculated
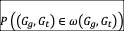
 for the three possible subsets above.

The central equation in the Caveman algorithm represents the probability of each joint genotype given the data
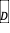
 and is calculated using the Bayes’ rule:


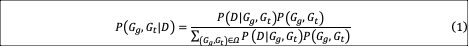


We have already discussed how the prior is calculated, it now remains to calculate
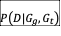
. We assume that for a given mapped read at position
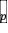
 the true base
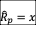
 is called as base
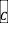
 with probability
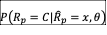
 given covariates
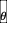
. Caveman considers the following covariates:

• lane/read group

• read order

• strand

• mapping quality

• base quality

• read position (position within read)

It is worth noting that the first 4 covariates are defined at the level of the read and the last 2 covariates at the base call level.

The relevant data,
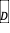
, is assumed to be the pileup of reads at the specified genomic position for both the normal sample
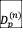
 and the tumour sample
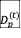
. If we assume that errors in the called base are conditionally independent given the covariates then we can write:


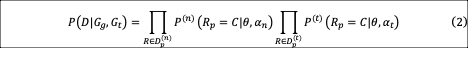


Now for a given sample,
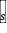
 (in our case
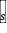
 is either ‘normal’ or ‘tumour’), that is a combination of germline sample and tumour sample with aberrant cell fraction
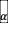
 we have:


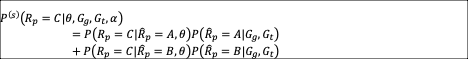


We adjust the aberrant cell fraction to account for different assumed copy number between germline and tumour,
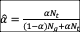
, so that it represents the probability that a read from
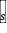
 comes from a tumour cell. For genotype,
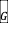
, let
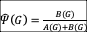
 represent the proportion of variant alleles in genotype
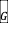
 which can then be adjusted for reference bias;
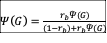
 so that it represents the probability that a given overlapping read from a cell that has genotype
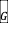
 carries the variant read (
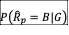
).

Then:


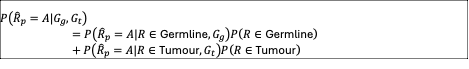


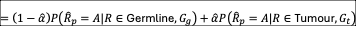


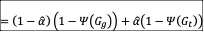


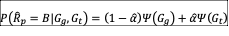


Substituting this into () and rearranging we have:


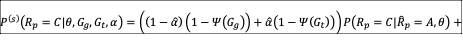


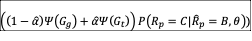


We now have nearly all the information required to calculate (2) and therefore (1). It remains only to find an expression for the so called profile probabilities
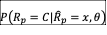
.

Recall that at each position
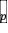
 in the genome we have a set of overlapping reads
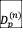
 and
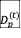
 for normal and tumour respectively. Now each read
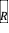
 at position
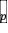
 has covariates
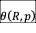
 and we form an overall count
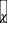
 of the number of times that each covariate appears across the genome where the called base is
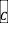
 and the true base is
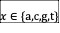


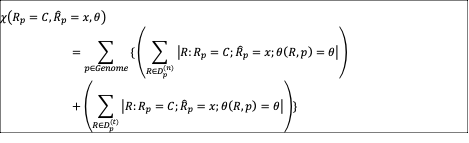


This counts matrix is then converted into an empirical estimate of the profile probabilities:


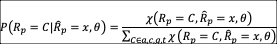


This process is only suitable where the number of erroneous non-reference base calls is much higher than the true number of non-reference base calls. Note in our implementation we exclude common SNP sites from the above calculation.
